# A Novel Zn_2_-Cys_6_ Transcription Factor AtrR Plays a Key Role in an Azole Resistance Mechanism of *Aspergillus fumigatus* by Co-regulating *cyp51A* and *cdr1B* Expressions

**DOI:** 10.1371/journal.ppat.1006096

**Published:** 2017-01-04

**Authors:** Daisuke Hagiwara, Daisuke Miura, Kiminori Shimizu, Sanjoy Paul, Ayumi Ohba, Tohru Gonoi, Akira Watanabe, Katsuhiko Kamei, Takahiro Shintani, W. Scott Moye-Rowley, Susumu Kawamoto, Katsuya Gomi

**Affiliations:** 1 Medical Mycology Research Center, Chiba University, Chiba, Japan; 2 Department of Bioindustrial Informatics and Genomics, Graduate School of Agricultural Science, Tohoku University, Sendai, Japan; 3 Department of Molecular Physiology and Biophysics, Carver College of Medicine, University of Iowa, Iowa City, Iowa, United States of America; Geisel School of Medicine at Dartmouth, UNITED STATES

## Abstract

Successful treatment of aspergillosis caused by *Aspergillus fumigatus* is threatened by an increasing incidence of drug resistance. This situation is further complicated by the finding that strains resistant to azoles, the major antifungal drugs for aspergillosis, have been widely disseminated across the globe. To elucidate mechanisms underlying azole resistance, we identified a novel transcription factor that is required for normal azole resistance in *Aspergillus* fungi including *A*. *fumigatus*, *Aspergillus oryzae*, and *Aspergillus nidulans*. This fungal-specific Zn_2_-Cys_6_ type transcription factor AtrR was found to regulate expression of the genes related to ergosterol biosynthesis, including *cyp51A* that encodes a target protein of azoles. The *atrR* deletion mutant showed impaired growth under hypoxic conditions and attenuation of virulence in murine infection model for aspergillosis. These results were similar to the phenotypes for a mutant strain lacking SrbA that is also a direct regulator for the *cyp51A* gene. Notably, AtrR was responsible for the expression of *cdr1B* that encodes an ABC transporter related to azole resistance, whereas SrbA was not involved in the regulation. Chromatin immunoprecipitation assays indicated that AtrR directly bound both the *cyp51A* and *cdr1B* promoters. In the clinically isolated itraconazole resistant strain that harbors a mutant Cyp51A (G54E), deletion of the *atrR* gene resulted in a hypersensitivity to the azole drugs. Together, our results revealed that AtrR plays a pivotal role in a novel azole resistance mechanism by co-regulating the drug target (Cyp51A) and putative drug efflux pump (Cdr1B).

## Author Summary

Better survival of chronically ill patients has produced an increased number of cases involving fungal infectious disease. This is partly due to a larger number of immunocompromised patients. Meanwhile, the catalogue of antifungal drugs remains quite limited and the appearance of resistant isolates is becoming more common. The filamentous fungus *Aspergillus fumigatus* is the main causative pathogen for deep-seated aspergillosis. For this fungus, resistance to azoles, which are the most commonly utilized drug for treatment of aspergillosis, has exhibited an alarming increase linked with elevated mortality. We know relatively little of the molecular details underpinning azole resistance in *A*. *fumigatus* other than alterations in the target enzyme. Here, we identified and characterized a novel transcription factor AtrR that is required for wild-type azole resistance in this fungus as well as other *Aspergillus* fungi, such as *Aspergillus oryzae* and *Aspergillus nidulans*. We discovered that AtrR co-regulates the target and pump, both of which are essential for azole resistance. More importantly, deletion of the *atrR* gene could sensitize a clinically isolated multi-azole resistant strain and exhibited a virulence defect in a mouse model. This success in overcoming an existing resistance mutation provides a new avenue for the prevention and treatment of aspergillosis.

## Introduction

Aspergillosis is one of the most common infectious diseases by mold, with *Aspergillus fumigatus* being the most frequently causative fungus with high mortality (more than 60%). Despite the importance of this disease, approved antifungals for treatment of aspergillosis are quite limited. Together with difficulties in early diagnosis, today aspergillosis is a major threat especially for immunocompromised patients. Further worsening the outlook for successful antifungal therapy, azole resistant *A*. *fumigatus* strains have been found with increasing incidence worldwide [1,2]. Azoles are an essential antifungal drug for therapy for chronic aspergillosis. In fact, azole resistant *A*. *fumigatus* strains exhibit a poor clinical outcome [3]. Therefore it is an urgent issue to unravel the molecular mechanisms underlying azole resistance and to develop new antifungal drugs or agents that are able to reverse azole resistance.

Mutations in the *cyp51A* gene locus have been frequently reported in azole resistant *A*. *fumigatus* strains. This locus encodes lanosterol-α-14-demethylase, the target enzyme of azole drugs [1,3]. Inhibition of the Cyp51A enzyme results in a significant change in sterol profile in *A*. *fumigatus* cells, where ergosterol deficiency as well as accumulation of toxic intermediates is thought to cause the antifungal effect [4,5]. Several reports have revealed that G54 and M220 are hot spots for azole resistance mutations in the Cyp51A protein [6]. Resistant mutations tend to arise during prolonged treatment of chronic aspergillosis with azole drugs [3,7,8]. In addition to resistance acquired during therapy, environmentally-derived resistance mutations have emerged as a major source of resistant organisms. Azole resistant strains with a combination of a tandem repeat in the promoter region of *cyp51A* and amino acid mutation(s) (TR34/L98H and TR46/Y121F/T289A) have been isolated from patients regardless of azole therapy history [9–11]. This pre-acquired azole resistant strain limits the alternatives for effective drug therapy of aspergillosis. Recently emerging mutations were described worldwide and range across isolates from Europe [10–13], Asia [14–16], and North America [17].

To date, one surveillance report provided data that azole resistant strains were up to 38% of the isolates from aspergillosis patients in the Netherlands, about 90% of which are related to Cyp51A [18]. Apart from alterations in *cyp51A*, a mutation in *hapE* [19] and overexpression of *cdr1B* [20] or *cyp51B* [21] were reported to be responsible for azole resistance in a clinical setting. Importantly, *cdr1B* encodes an ATP-binding cassette (ABC) transporter sharing high sequence similarity with *Saccharomyces cerevisiae* Pdr15 and Pdr5, *Candida albicans* Cdr4 and Cdr1, and *Candida glabrata* Cdr1 and Pdh1. This shared homology is suggestive of a role of Cdr1B in drug efflux. The fact that deletion of the *cdr1B* resulted in azole sensitive phenotype further supported the important role in azole resistance in *A*. *fumigatus* [20,22].

The transcription factors involved in azole resistance in fungi have been intensively investigated in *S*. *cerevisiae*. The well-studied Pdr1 and its paralog, Pdr3, are zinc finger transcription factors that regulate the pleiotropic drug response in the yeast [23]. Pdr1 and Pdr3 serve as transcriptional activators and repressors by binding to pleiotropic drug response elements (PDREs) that are found in the promoter regions of target genes [24]. The targets include the ABC transporters encoded by *PDR5*, *PDR10*, and *PDR15* [25].

In filamentous fungi including plant and animal pathogens, only one regulatory protein involved in azole resistance has been studied. This protein was designated SrbA and encodes a homologue of the Sterol Regulatory Element-Binding Proteins (SREBPs). The SREBP is a transcription factor with a basic Helix-Loop-Helix (bHLH) DNA-binding domain and is well conserved in mammalian cells, where it regulates cholesterol synthesis and uptake [26]. Roles of the *A*. *fumigatus* SREBP, SrbA, were recently demonstrated [27,28]. SrbA was shown to regulate expression of multiple genes related to ergosterol biosynthesis pathway, including *erg3*, *erg24A*, and *erg25A*. A mutant strain defective in *srbA* showed hypersensitivity to azoles. Further experiments revealed that SrbA exerts a direct regulation on these genes, suggesting that this factor is a central regulator of ergosterol biosynthesis in the fungus [29,30]. Importantly, the *srbA* mutant was unable to grow under hypoxic condition. This is because SREBPs sense sterol level in the cells as an indirect indicator of oxygen. The *srbA* mutant was unable to adapt to hypoxic conditions that the fungi are thought to encounter at infection sites. This inability of the mutant to grow under hypoxia is also thought to lead to a strong attenuation in virulence in a mouse infection model. Whereas *Candida* species have no obvious SREBP orthologs, *Cryptococcus neoformans* has a SREBP, Sre1, which is also involved in azole sensitivity, hypoxia growth, and virulence [31,32]. These findings highlighted that ergosterol biosynthesis is crucial for not only drug resistance but also for adaptation to hypoxia and virulence. Besides SREBPs, however, current knowledge about the transcription factors responsible for azole resistance and sterol biosynthesis is limited particularly in pathogenic fungi.

In the present study, we identified a novel transcription factor involved in azole resistance mechanism in *Aspergillus* fungi. From a genetic screen in *Aspergillus oryzae* designed to identify transcriptional regulators of azole drug resistance, we found a novel Gal4-type Zn_2_-Cys_6_ zinc finger domain-containing transcription factor designated AtrR. Deletion of the *atrR* gene in *A*. *oryzae* and *Aspergillus nidulans* resulted in hypersensitivity to azole drugs. Similarly, deletion of the *atrR* gene in *A*. *fumigatus* resulted in hypersensitivity to azole antifungals, inability to grow under hypoxic conditions, attenuated virulence, and decreased expression of *cyp51A* as well as the ABC transporter-encoding gene *cdr1B*. Even in a clinical azole resistant strain (*cyp51A* G54E), deletion of *atrR* led to an azole-hypersensitive phenotype. All our data indicate that AtrR plays an essential role in azole resistance in *A*. *fumigatus*. Importantly, this is the first identification of a regulatory protein coordinating expression of not only an azole drug target (Cyp51A) but also for the putative drug efflux transporter (Cdr1B).

## Results

### Identification of *Aspergillus oryzae* AtrR transcription factor responsible for azole resistance

**Fig 1:**
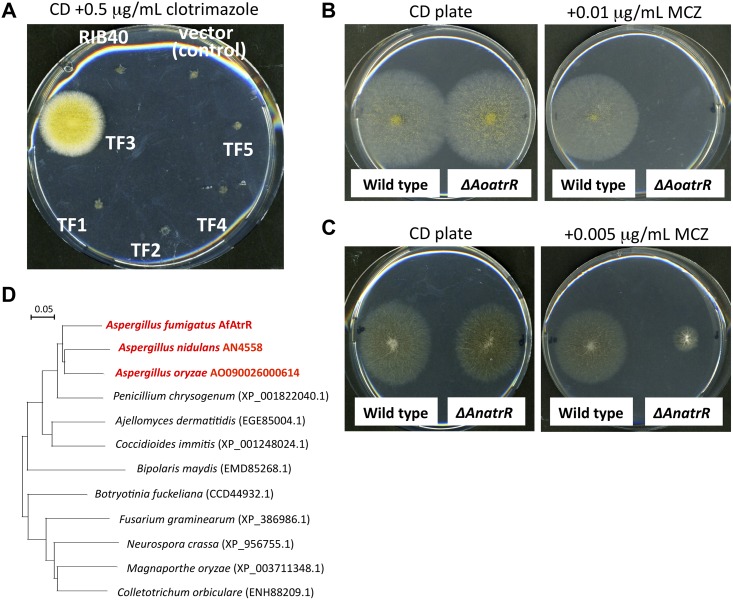
Identification of AtrR-family genes in *A*. *oryzae* and *A*. *nidulans*. (A) Growth of *A*. *oryzae* strains overexpressing the candidate genes on medium plate in the presence of clotrimazole. 10^2^ conidia of the strains were inoculated on CD plate (1% maltose) containing 0.5 μg/ml clotrimazole and incubate at 30°C for 7 days. (B) Growth of the *AoatrR* deletion strain on medium plate in the presence of miconazole. 10^2^ conidia of *A*. *oryzae* LigD (wild type) and the *AoatrR* deletion mutant (*ΔAoatrR*) were inoculated on CD plate (1% glucose) containing 0.01 μg/ml miconazole (MCZ) and incubate at 30°C for 5 days. (C) Growth of the *AnatrR* deletion strain on medium plate in the presence of MCZ. 10^2^ conidia of *A*. *nidulans* KU70 (wild type) and the *AnatrR* deletion mutant (*ΔAnatrR*) were inoculated on CD plate (1% glucose) containing 0.005 μg/ml miconazole and incubate at 37°C for 5 days. (D) Phylogenetic tree of fungal AtrR ortholog proteins. The protein sequences were retrieved from NCBI database, and the sequence IDs were shown in brackets. The sequences were aligned by Clustal W program and the distances were calculated. The phylogenetic tree was drawn with an NJplot program.

To identify the transcription factors (TFs) responsible for azole resistance in filamentous fungi, we utilized a transcription factor-overexpressing library of *Aspergillus oryzae* that was previously constructed in the Noda Institute for Scientific Research [33] First, we searched for *A*. *oryzae* transcription factors with a DNA-binding domain related to *S*. *cerevisiae* Pdr1 and Pdr3, resulting in 5 candidate proteins (designated TF1 to TF5) (S1A Fig). To evaluate a role of these TFs in azole resistance, strains overexpressing each TF were tested for growth in the presence of azoles. Only *A*. *oryzae* strain expressing TF3 showed a significant hyper-resistance to clotrimazole (Fig 1A). TF3 corresponds to AO090026000614 which is a protein with 894 amino acids containing two characteristic motifs designated as GAL4-like Zn(II)_2_/Cys_6_ binuclear cluster DNA-binding domains (IPR001138) and a fungal specific transcription factor domain (IPR007219) (S1B Fig). Based on the screening design to find transcription factors homologous to ScPdr1 and/or ScPdr3 that regulate ABC transporter gene expression, we named this novel TF AoAtrR (*A*. *oryzae* ABC-transporter regulating transcription factor). To further characterize the role of AoAtrR, the corresponding gene was deleted in *A*. *oryzae* host strain (RIB40) (S2A and S2B Fig), and azole resistance was examined. The *AoatrR* mutant showed hyper-sensitivity in the presence of 0.01 μg/mL miconazole, whereas the host strain was able to grow under this condition (Fig 1B). These results indicated that AoAtrR is involved in azole resistance in *A*. *oryzae*. The closely related species *Aspergillus nidulans* has an orthologous protein (designated AnAtrR; amino acids sequence identity: 79.9%) (S1B Fig), and the *AnatrR* deletion mutant strain (S2C and S2D Fig) also clearly exhibited azole sensitive phenotype (Fig 1C). As a result, the AtrR-family protein appears to be a novel TF responsible for azole resistance mechanisms in *Aspergillus* species. This AtrR-family TF is widely conserved in filamentous fungi including important plant pathogenic fungi (*Fusarium graminearum*, *Magnaporthe oryzae*, and *Collectotrichum orbiculare*) and human pathogenic fungi (*Aspergillus fumigatus*, *Ajellomyces dermatitidis*, and *Coccidioides immitis*) (Fig 1D).

### AtrR is involved in azole resistance of *A*. *fumigatus*

**Fig 2:**
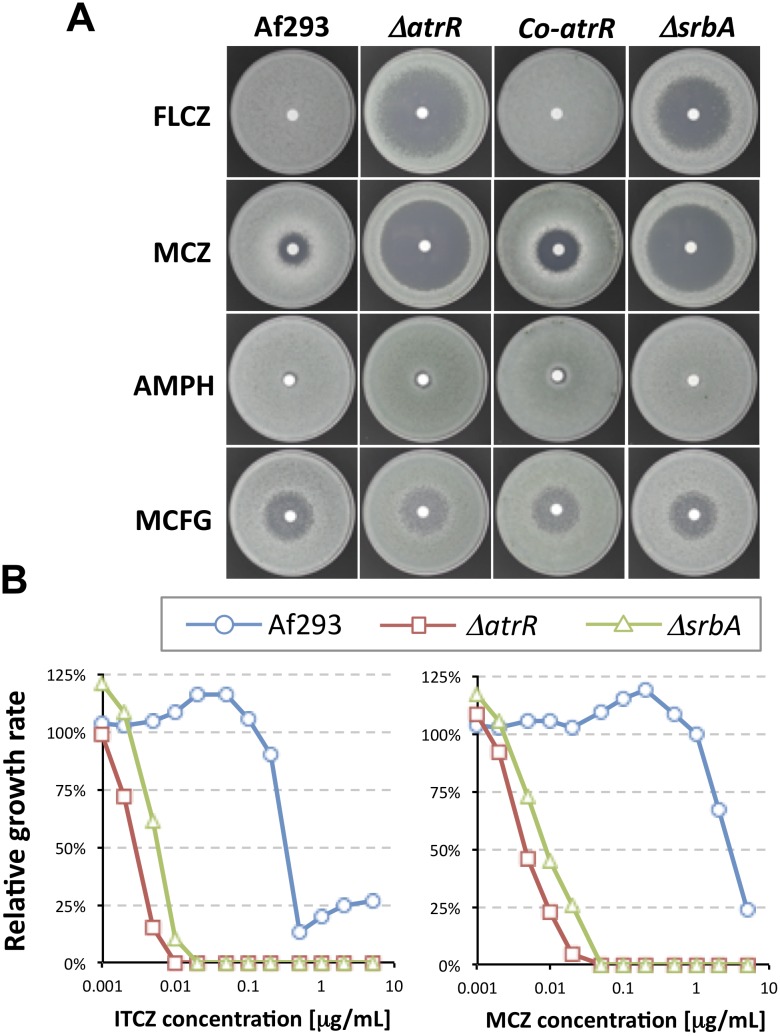
Drug susceptibility of *A*. *fumigatus* strains linked to modifications of the *atrR* and *srbA* genes. (A) Antifungal drug susceptibility test by paper-disc diffusion assay. GMM plates containing conidia of each strain were prepared. A paper-disc was placed on center of the plate, and 10 μl of drug solution indicated was dropped on it (fluconazole (FLCZ): 10 mg/mL; MCZ: 10 mg/mL; amphotericin B (AMPH): 0.25 mg/mL; micafungin (MCFG): 0.02 mg/mL). The plates were incubated for 48 h before photographed. (B) Inhibition test for colony growth by antifungal drugs. The conidia of each strain were center-inoculated onto GMM plates containing indicated concentrations of itraconazole (ITCZ) or MCZ. The plates were incubated for 70 h. The colony diameter was measured in triplicates, and the mean values for plates with drugs were compared with those without drugs. The percentage of growth was plotted in the graphs.

To expand our understanding of molecular mechanism underlying azole resistance with clinically important fungi, we searched for an AtrR homologue in the human pathogenic fungus *A*. *fumigatus*. The AfAtrR in *A*. *fumigatus* (hereafter, designated AtrR to simplify descriptions) is homologous to AoAtrR and AnAtrR, and the deletion mutant and the complementing strains (designated *ΔatrR* and *Co-atrR*) were constructed in the *A*. *fumigatus* host strain (Af293) (S3 Fig). A disc-diffusion assay revealed that *ΔatrR* was hypersensitive to fluconazole and miconazole, whereas the mutant showed no distinguishable sensitivity to amphotericin B and micafungin (Fig 2A). In addition to the medical azoles, *ΔatrR* showed hypersensitivity to widely used azole fungicides, bromuconazole, tebuconazole, difenoconazole, and propiconazole (S4 Fig). Growth inhibition assay with varied concentrations of drugs in plate media indicated that *ΔatrR* was highly susceptible to itraconazole and miconazole (Fig 2B). The complemented strain showed comparable drug sensitivity to the WT, indicating that these phenotypes were caused by deletion of the *atrR* gene.

### AtrR regulates *cyp51A* expression

**Fig 3:**
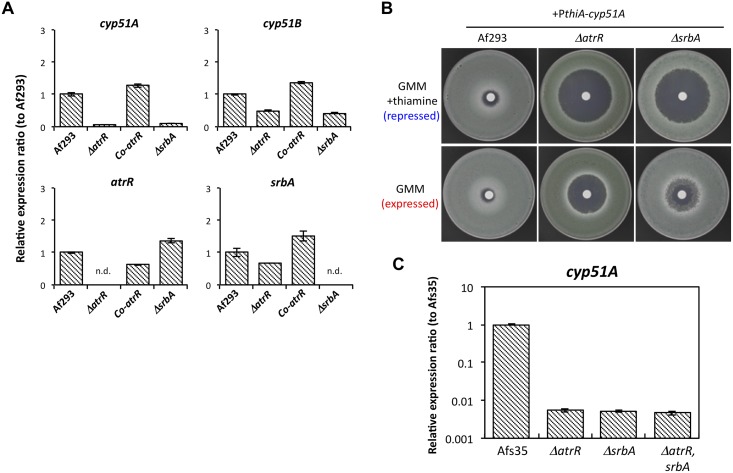
Expression analysis in *atrR* and *srbA* deletion mutants. (A) Expression levels of the *cyp51A* and *cyp51B* as well as the *atrR* and *srbA* genes were determined by real-time RT-PCR method. The strains were cultivated in GMM for 24 h. The expression levels of gene of interest were normalized with that of actin gene. Relative expression ratios against Af293 (WT) are shown. Error bars represent the standard deviations based on three independent replicates. (B) MCZ susceptibility test for the Af293, *ΔatrR*, and *ΔsrbA* strains carrying a *cyp51A* gene fused with a *thiA* promoter. MCZ susceptibility was examined as described above. When thiamine was present, expression of the functional *cyp51A* mRNA driven by *thiA* promoter was repressed. The plates were incubated for 48 h before photographed. (C) Expression levels of *cyp51A* were determined by real-time RT-PCR in the mutant strains of *ΔatrR*, *ΔsrbA*, *ΔatrR ΔsrbA* of Afs35-background. The strains were cultivated in YGMM for 18 h.

We assumed that hypersensitivity to azoles in *ΔatrR* was associated with a defect in expression of the target protein Cyp51A. In fact, the expression level of *cyp51A* gene was quite low in the *ΔatrR* strain (less than 5% of that in the WT), whereas *cyp51B* expression was also reduced (approximately 50% of the WT) (Fig 3A). Since SrbA was previously characterized to regulate *cyp51A* [29], we also constructed a null mutant of *srbA* and compared the two mutants (*ΔatrR* and *ΔsrbA*) for their relative effects on *cyp51A* and *cyp51B* expression levels. We found that the expression levels of *cyp51A* and *cyp51B* were largely comparable between *ΔsrbA* and *ΔatrR* (Fig 3A). Although not large, there was a slight increase in expression level for *srbA* in *ΔatrR* mutant and a slight decrease in *atrR* expression in *ΔsrbA*, suggesting effects of AtrR and SrbA on each other’s mRNA expression (Fig 3A). To determine if the heterologous expression of *cyp51A* was sufficient to restore azole resistance in mutants lacking either *atrR* or *srbA*, we introduced an ectopic *cyp51A* gene regulated by the *thiA* promoter (P*thiA*-*cyp51A*) into the *ΔatrR* and *ΔsrbA* backgrounds. When the *thiA* promoter was maximally active (absence of thiamine), the resulting expression level of Cyp51A was able to partially suppress loss of either transcription factor gene in both strains. *ΔatrR*+P*thiA*-*cyp51A* and *ΔsrbA*+P*thiA*-*cyp51A* showed smaller inhibition halos against miconazole compared to those under the repressed condition (presence of thiamine) (Fig 3B). This suggested that azole susceptibility in *ΔatrR* and *ΔsrbA* are partly a result of decreased *cyp51A* expression.

Hypersensitivities to fluconazole, miconazole, and itraconazole were also seen in the *ΔsrbA* strain (Fig 2A and 2B). The *ΔatrR* strain was slightly more sensitive to fluconazole and itraconazole compared to a mutant lacking the *srbA* gene. However, both of these factors are required for normal azole resistance in *A*. *fumigatus*. In order to confirm the relative role of these genes across strains, these mutations were produced in a different genetic background (Afs35). The double *ΔatrR ΔsrbA* mutation was also produced in this same strain. The expression level of *cyp51A* was comparable between the *ΔatrR*, *ΔsrbA*, as well as *ΔatrR ΔsrbA*, supporting the required function of both AtrR and SrbA (Fig 3C).

### AtrR regulates genes related to ergosterol biosynthesis

**Fig 4:**
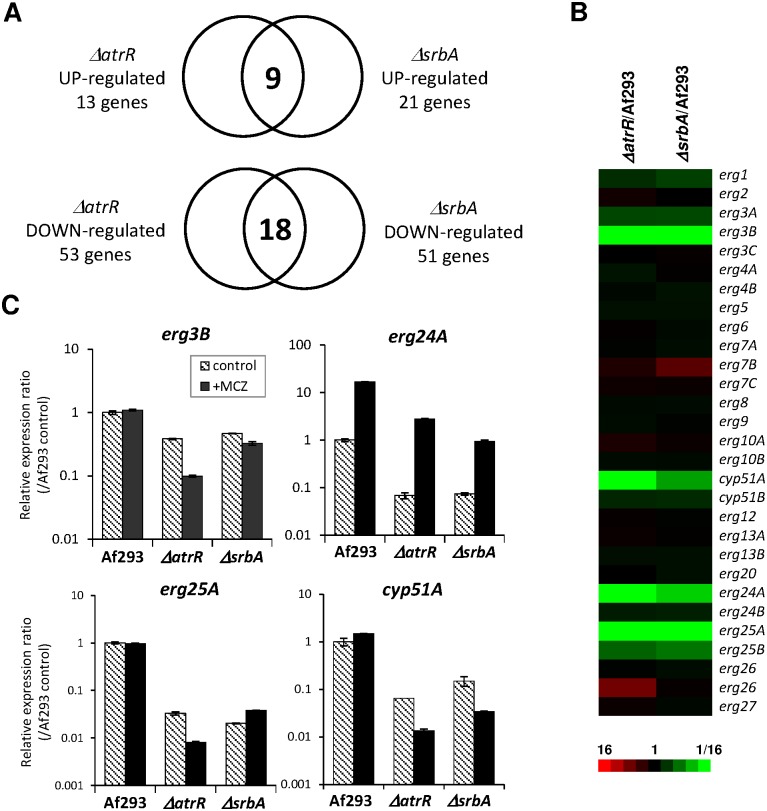
AtrR and SrbA regulate genes associated with the ergosterol biosynthesis pathway. (A) The AtrR- and SrbA-dependent genes determined by RNA-sequencing analysis. Numbers of gene with more than 4-fold or less than 1/4-fold change in expression level compared to Af293 are shown. The expression level of nine genes in *ΔatrR* and *ΔsrbA* strains was more than 4-fold of Af293, and the expression level of eighteen genes in *ΔatrR* and *ΔsrbA* strains was less than 1/4-fold of Af293. (B) Heat map of expression of the *erg* genes. The levels of expression ratio were shown. (C) Expression levels of *erg3B*, *erg24A*, *erg25A*, and *cyp51A* genes in response to MCZ addition were determined by real-time RT PCR method. The strains were cultivated in GMM for 24 h followed by 2 h culture with MCZ (final concentration, 2 μg/mL) or without MCZ (control). The expression levels of gene of interest were normalized with that of actin gene. Relative expression ratios against Af293 (WT without MCZ) are shown. Error bars represent the standard deviations based on three independent replicates.

To globally identify the genes responsive to AtrR, we performed transcriptome analysis using RNA-sequencing. Compared to the WT, 13 and 53 genes were up-regulated and down-regulated, respectively, in the *ΔatrR* strain, showing AtrR-dependent genes. We also determined SrbA-dependent genes in a similar fashion, resulting in 21 up-regulated genes and 51 down-regulated genes. Comparing each group of up- and down-regulated genes between the *ΔatrR* and *ΔsrbA* strains, we found that 9 genes were commonly negatively regulated by AtrR and SrbA whereas 18 genes were positively regulated (Fig 4A, Table 1, S1 and S2 Tables). This comparative transcriptome analysis revealed that AtrR and SrbA were required for the expression of *erg3B*, *erg24A*, and *erg25A* as well as *cyp51A* genes, all of which are related to ergosterol biosynthesis. Interestingly, the other genes related to ergosterol biosynthesis were only modestly affected by deletion of either *atrR* or *srbA* genes (Fig 4B). Involvement of AtrR and SrbA in the transcriptional regulation of the *erg3B*, *erg24A*, *erg25A*, and *cyp51A* genes was further verified by real-time RT PCR analysis (Fig 4C). As a previous report demonstrated that SrbA regulates genes related to the ergosterol biosynthesis pathway [30], our data indicate that AtrR also functions in regulating this pathway.

**Table 1:** RNA-sequencing expression data for the genes regulated by AtrR and SrbA.

			RPKM			Expression ratio		
	ID	Gene name	Af293	*ΔatrR*	*ΔsrbA*	*ΔatrR*/Af293	*ΔsrbA*/Af293	Annotation
**UP in *ΔatrR* and *ΔsrbA***								
	Afu1g03800		13.8	83.1	205.3	6.01	14.84	C6 transcription factor, putative
	Afu2g05060	*aoxA*	474.7	4255.6	6685.0	8.97	14.08	alternative oxidase AlxA, putative
	Afu3g03280		46.7	324.7	542.2	6.95	11.60	FAD binding monooxygenase, putative
	Afu3g13010		52.5	390.8	567.2	7.44	10.80	Zn-dependent hydrolase/oxidoreductase family protein, putative
	Afu4g09450		24.0	97.0	171.1	4.04	7.13	hypothetical protein
	Afu4g14380		203.2	1313.3	1894.0	6.46	9.32	hypothetical protein
	Afu5g06070	*mdr1*	11.9	65.9	108.2	5.53	9.09	ABC multidrug transporter Mdr1
	Afu7g00950		10.3	60.5	77.7	5.87	7.54	MFS monosaccharide transporter, putative
	Afu8g01860		30.6	163.0	218.8	5.32	7.15	hypothetical protein
**DOWN in *ΔatrR* and *ΔsrbA***								
	Afu1g03150	*erg24A*	25.5	1.1	2.5	0.04	0.10	c-14 sterol reductase
	Afu2g00320	*erg3B*	116.8	0.4	0.8	0.00	0.01	sterol delta 5,6-desaturase, putative
	Afu2g00330		10.5	1.5	0.6	0.15	0.06	beta-alanine synthase, putative
	Afu2g17660		11.7	1.5	0.8	0.13	0.07	C4-dicarboxylate transporter/malic acid transport protein, putative
	Afu3g00810	*hyd1*	39.7	1.2	0.0	0.03	0.00	cholestenol delta-isomerase, putative
	Afu3g00820		14.5	0.3	1.0	0.02	0.07	conserved hypothetical protein
	Afu4g06890	*cyp51A*	261.4	14.9	40.2	0.06	0.15	14-alpha sterol demethylase Cyp51A
	Afu5g01248		24.5	0.8	0.8	0.03	0.03	conserved hypothetical protein
	Afu5g14740	*fleA*	91.6	9.3	6.7	0.10	0.07	fucose-specific lectin FleA
	Afu7g01490	*ptr2*	149.7	23.4	27.8	0.16	0.19	MFS peptide transporter Ptr2, putative
	Afu704920		138.3	20.1	16.6	0.15	0.12	hypothetical protein
	Afu7g04930	*pr1*	26.7	0.7	2.2	0.03	0.08	alkaline serine protease (PR1)/allergen F18-like
	Afu7g06981		13.0	1.5	2.5	0.12	0.19	conserved hypothetical protein
	Afu8g00830		135.7	13.2	23.9	0.10	0.18	conserved hypothetical protein
	Afu8g01330		10.2	0.6	1.8	0.06	0.18	hypothetical protein
	Afu8g02440	*erg25A*	1345.1	84.5	40.5	0.06	0.03	C-4 methyl sterol oxidase, putative
	Afu8g06000		18.2	4.3	4.3	0.24	0.24	FMN dependent dehydrogenase, putative
	Afu8g06010		36.8	6.4	8.8	0.17	0.24	C6 transcription factor, putative

### AtrR is necessary for hypoxia adaptation and virulence

**Fig 5:**
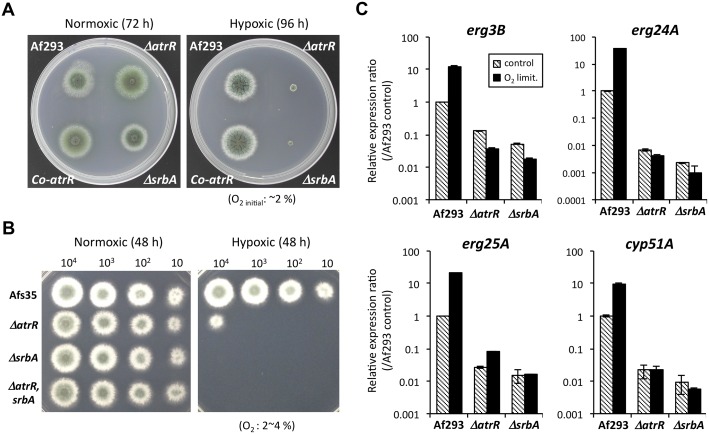
AtrR is involved in adaptation to hypoxia. (A) Growth test under normoxic and hypoxic conditions. The conidia of each strain were inoculated on GMM plate, and the plate was incubated in anaeropack system (a sealed pack) where initial concentration of oxygen was set at 2% (hypoxia condition). After 96 h, oxygen in the pack appeared to be completely consumed as estimated from no more growth of all colonies on the plate. The plate for normoxia was incubated in a regular incubator (21% oxygen) for 72 h. (B) Growth of Afs35-background strains under normoxic and hypoxic conditions. The 10 to 10^4^ conidia of each strain were inoculated on GMM plate, and the plate was incubated in anaeropack system (a sealed pack) for 48 h, where concentration of oxygen was maintained at 2 to 4% (moderate hypoxia condition). (C) Expression levels of *erg3B*, *erg24A*, *erg25A*, and *cyp51A* genes in response to oxygen limitation (O_2_ limit.) were determined by real-time RT PCR method. The strains were cultivated in YGMM for 18 h. A half potion of the culture was transferred into a 50 mL tube with a screwed cap. The tube was closed with a cap carefully eliminating air, and was incubated for another 1 h. The expression levels of gene of interest were normalized with that of actin gene. Relative expression ratios against Af293 (WT before oxygen limitation) are shown. Error bars represent the standard deviations based on three independent replicates.

As both AtrR and SrbA have a role in transcriptional regulation of the ergosterol biosynthesis pathway, we wanted to assess if AtrR also contributed to hypoxic growth and virulence as previously shown for SrbA [27,28]. We tested growth under hypoxic conditions and found that growth of both *ΔatrR* and *ΔsrbA* mutants, constructed in either the Af293 or Afs35 genetic backgrounds, was strongly inhibited under this condition (Fig 5A and 5B). These results indicate a crucial role for AtrR in adaptation to hypoxia. To confirm the participation of AtrR in the hypoxic response, the transcriptional regulation of *erg3B*, *erg24A*, *erg25A*, and *cyp51A* upon oxygen limitation (1 h) was compared in *ΔatrR* and *ΔsrbA* strains. The induction of these genes upon oxygen limitation was diminished or eliminated in the both mutants (Fig 5C). These results indicated that AtrR also plays an important role in hypoxia adaptation in *A*. *fumigatus* physiology as found for SrbA.

**Fig 6:**
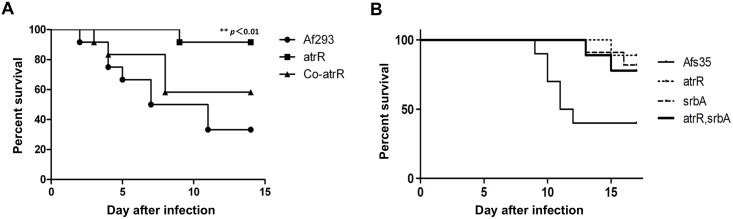
AtrR is involved in *A*. *fumigatus* virulence. (A) Mouse infection test with the Af293-background strains. Outbred ICR mice (n = 12) were immune-compromised by intraperitoneally injected cyclophosphamide (200 mg/kg) at Day -4, -2, 1, 3, 5, and 7. Mice were infected intratracheally with 2.5×10^7^ conidia in a volume of 25 μL of each strain (Af293, *ΔatrR*, and *Co-atrR*). Statistical significance was examined by log-rank test. P value for comparison between *ΔatrR* and Af293 was lower than 0.01. (B) Mouse infection test with the Afs35-background strains. Outbred ICR mice (n = 9 to 11) were immune-compromised by intraperitoneally injected cyclophosphamide (150 mg/kg) at Day -4, -1, 3, 6, 9, and 12. Cortisone acetate was also administrated subcutaneously at a concentration of 200 mg per kg of body weight on day -1. Mice were infected intratracheally with 3×10^5^ conidia in a volume of 30 μL of each strain (Afs35, *ΔatrR*, *ΔsrbA*, and *ΔatrR ΔsrbA*) on Day 0.

We also examined the role of AtrR in pathogenesis using a murine infection model. In this model for aspergillosis, virulence of the *ΔatrR* strain was significantly reduced compared to the WT and *Co-atrR* strains (Fig 6A). The reduction in virulence was also seen in the Afs35-background *ΔatrR* as well as *ΔsrbA* and *ΔatrR ΔsrbA* (Fig 6B). These results indicated that AtrR was required for full pathogenicity of *A*. *fumigatus*. This is again reminiscent of the role of SrbA in virulence, where *ΔsrbA* showed significantly attenuated virulence in immune-compromised mice [27].

### AtrR is a regulator of ABC transporter gene *cdr1B*

**Fig 7:**
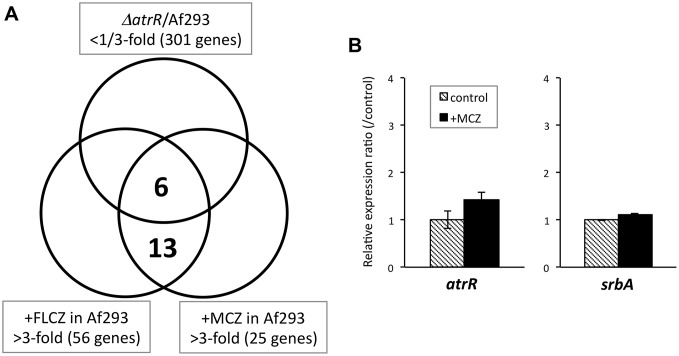
FLCZ- and MCZ-responsive and AtrR-dependent genes determined by RNA-sequencing analysis. (A) The *ΔatrR* and Af293 strains were cultivated in PDB for 20 h followed by FLCZ (final concentration, 64 μg/mL), MCZ (2 μg/mL), or DMSO (as a control) treatment for 2 h. Number of gene with less than 1/3-fold change in expression level in *ΔatrR* (DMSO) compared to Af293 (DMSO) was shown. Numbers of gene with more than 3-fold upregulation upon FLCZ or MCZ treatment (2 h) in Af293 were shown. Expression of six genes was induced upon FLCZ and MCZ treatment and these genes were dependent on AtrR, and expression of thirteen genes was induced upon FLCZ and MCZ treatment and these genes were independent on AtrR. (B) Expression level of *atrR* and *srbA* genes in response to MCZ addition was determined by real-time RT PCR method. The Af293 strain was cultivated in GMM for 24 h followed by 2 h culture with MCZ (final concentration, 2 μg/mL) or without MCZ (control). The expression levels of gene of interest were normalized with that of actin gene. Relative expression ratios against control (Af293 without MCZ) are shown. Error bars represent the standard deviations based on three independent replicates.

To determine the role of AtrR in drug-induced gene expression in the *A*. *fumigatus* azole response, we performed further RNA-sequencing analysis following azole challenges. WT and *ΔatrR* were cultivated in PDB for 20 h, and then fluconazole (final concentration, 64 μg/mL) or miconazole (2 μg/mL) were added. After 2 h, the mycelia were harvested, and the transcriptomes were determined by RNA-sequencing. Of 7015 genes that showed >10 reads per kilobase of exon per million mapped reads (RPKM) in WT without drug treatment, 301 genes were reduced to <33% in *ΔatrR* compared to the WT (Fig 7A). Upon fluconazole and miconazole treatment, 56 and 25 genes were upregulated in WT, respectively. Among them, 19 genes were commonly responsive to fluconazole and miconazole, 6 genes of which were dependent on AtrR. The azole-responsive AtrR-dependent genes included *cyp51A*, *erg24A*, *erg24B*, *erg25A* as well as *hyd1* and Afu3g00820. These results again confirmed that AtrR regulated multiple genes related to the ergosterol biosynthesis pathway (Table 2). While *atrR* and *srbA* expression was slightly affected in response to fluconazole (1.22- and 1.62-fold, respectively) and miconazole (1.27- and 1.37-fold) treatment, real-time RT-PCR analysis suggested that the effect of azoles on the expressions were only subtle (Fig 7B).

**Table 2:** RNA-sequencing analysis for Af293 and *ΔatrR* in response to azole drugs.

		RPKM									
		Af293			*ΔatrR*						
ID	Gene name	+DMSO	+FLCZ	+MCZ	+DMSO	+FLCZ	+MCZ	*ΔatrR* (DMSO) /Af293 (DMSO)	Af293 (FLCZ) /Af293 (DMSO)	Af293 (MCZ) /Af293 (DMSO)	Predicted function
**AtrR- and azole treatment-dependent genes**											
Afu4g06890	*cyp51A*	249.0	901.5	952.8	3.9	15.3	18.9	0.02	3.62	3.83	14-alpha sterol demethylase
Afu1g03150	*erg24A*	45.6	605.5	736.4	2.0	16.7	35.7	0.04	13.28	16.15	C-14 sterol reductase
Afu1g05720	*erg24B*	95.0	441.6	409.8	26.2	23.6	32.1	0.28	4.65	4.31	C-14 sterol reductase
Afu8g02440	*erg25A*	597.8	1965.1	1906.6	10.0	13.0	24.7	0.02	3.29	3.19	C-4 methyl sterol oxidase
Afu3g00810	*hyd1*	62.94	320.39	402.68	0.00	19.36	20.35	0.00	5.09	6.40	cholestenol delta-isomerase, putative
Afu3g00820		15.96	123.97	118.89	3.63	2.10	13.82	0.23	7.77	7.45	putative exported protein
**PDR sub-family ABC transporters**											
Afu1g14330	*cdr1B*	61.1	125.5	111.3	5.7	9.9	6.9	0.09	2.06	1.82	ABC transporter, putative
Afu1g17440	*abcA*	0.3	0.0	0.0	0.3	0.0	0.4	1.06	0.00	0.00	ABC drug exporter AbcA
Afu2g15130		24.0	36.1	28.4	24.8	22.2	14.9	1.03	1.50	1.18	ABC multidrug transporter, putative
Afu3g01400		10.6	26.7	10.1	27.6	112.7	59.7	2.59	2.51	0.95	ABC multidrug transporter, putative
Afu3g07300	*atrI*	9.7	11.0	11.1	18.5	20.2	18.7	1.91	1.14	1.15	ABC multidrug transporter, putative
Afu4g01050		0.4	3.1	0.0	0.4	5.2	3.8	1.06	8.79	0.00	ABC multidrug transporter, putative
Afu5g00790		2.6	11.3	10.1	6.8	17.8	60.1	2.65	4.39	3.91	ABC multidrug transporter, putative
Afu5g02260		5.2	5.3	2.7	4.1	6.0	3.1	0.80	1.03	0.52	ABC multidrug transporter, putative
Afu5g09460		29.4	20.7	18.6	37.6	23.6	24.8	1.28	0.70	0.63	ABC transporter, putative
Afu6g04360	*atrF*	3.4	3.7	1.9	8.5	6.8	3.0	2.51	1.10	0.57	ABC drug exporter AtrF
Afu6g08020		8.1	9.2	8.1	7.6	13.8	7.2	0.94	1.14	1.00	ABC transporter, putative
Afu8g02650		3.3	5.4	6.8	5.2	5.1	5.9	1.59	1.65	2.09	ABC multidrug transporter, putative
**Transcription factors**											
Afu5g09420	*ccg-8*	46.4	40.8	43.6	41.4	41.1	47.2	0.89	0.88	0.94	clock controled protein (Ccg-8), putative
Afu1g16460	*ads-4*	88.0	103.1	94.8	83.4	129.5	135.8	0.95	1.17	1.08	bZIP transcription factor (LziP), putative
**Members of SREBP pathway**											
Afu2g01260	*srbA*	59.0	95.3	80.5	37.7	27.0	30.8	0.64	1.62	1.37	HLH transcription factor, putative
Afu1g12080	*dscA*	23.0	18.8	16.0	21.9	26.2	14.3	0.95	0.82	0.70	RING finger ubiquitin ligase (Tul1), putative
Afu4g11680	*dscB*	70.6	60.4	65.5	28.3	43.5	53.3	0.40	0.85	0.93	conserved hypothetical protein
Afu4g10700	*dscC*	44.4	55.5	41.5	29.2	45.2	48.2	0.66	1.25	0.94	conserved membrane protein, putative
Afu3g08340	*dscD*	10.0	12.6	6.0	15.2	23.7	10.4	1.52	1.26	0.60	conserved hypothetical protein
Afu1g14320	*dscE*	47.9	61.7	75.9	52.8	56.9	59.2	1.10	1.29	1.58	UBX domain protein
Afu6g12750	*rbdB*(*rbdA*)	55.6	45.8	48.9	35.0	43.3	21.1	0.63	0.82	0.88	rhomboid family protein, putative
Afu6g02150	*sppA*	28.6	36.4	26.7	27.2	16.7	21.9	0.95	1.27	0.93	signal peptide peptidase, putative

**Fig 8:**
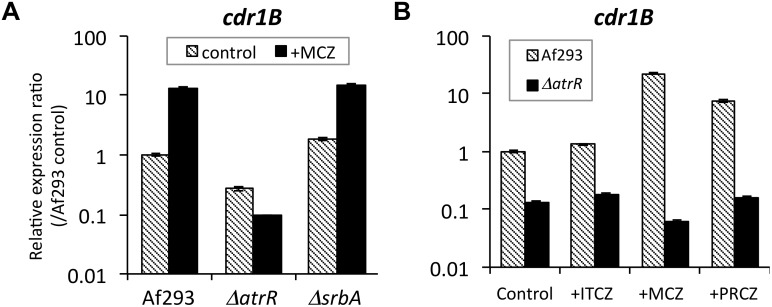
Expression levels of the *cdr1B* gene in response to azoles were determined by real-time RT PCR method. (A) AtrR is involved in regulating *cdr1B* expression. Af293, *ΔatrR*, and *ΔsrbA* strains were cultivated in GMM for 24 h followed by 2 h culture with or without MCZ (final concentration, 2 μg/mL). (B) Af293 and *ΔatrR* strains were cultivated in GMM for 24 h followed by 2h culture with ITCZ, MCZ, or propiconazole (PRCZ) (final concentrations, 2 μg/mL). The expression level of *cdr1B* was normalized with that of actin gene. Relative expression ratios against Af293 (WT without drug) are shown. Error bars represent the standard deviations based on three independent replicates.

By comparing amino acid sequences, *A*. *fumigatus* was found to possess 12 PDR-type ABC transporters. Among them, *cdr1B* expression was highest in WT according to the transcriptome data, whereas the expression level was reduced to less than 10% in the *ΔatrR* strain (Table 2). On the contrary, expressions of some transporter genes (i.e. Afu3g01400 and AtrI) were slightly higher in the *ΔatrR* compared to the WT, which suggests a negative role for AtrR. We then verified the expression profiles for the *cdr1B* by real-time RT PCR analysis, showing that the basal *cdr1B* expression was reduced in the *ΔatrR* strain (approximately 25% relative to the WT), and there was no induced expression in *ΔatrR* (Fig 8A). In a sharp contrast, the *ΔsrbA* strain showed WT-level basal expression and upregulation of *cdr1B*. This result clearly indicated that AtrR is responsible for *cdr1B* expression while SrbA is not involved in control of this transporter. Furthermore, we found that when treated with propiconazole, a representative of an azole fungicide, *cdr1B* expression was upregulated. This induction was also dependent on AtrR (Fig 8B) and supported a crucial role of AtrR in *cdr1B* expression in *A*. *fumigatus*.

### AtrR exerts direct regulation for *cyp51A* and *cdr1B*

**Fig 9:**
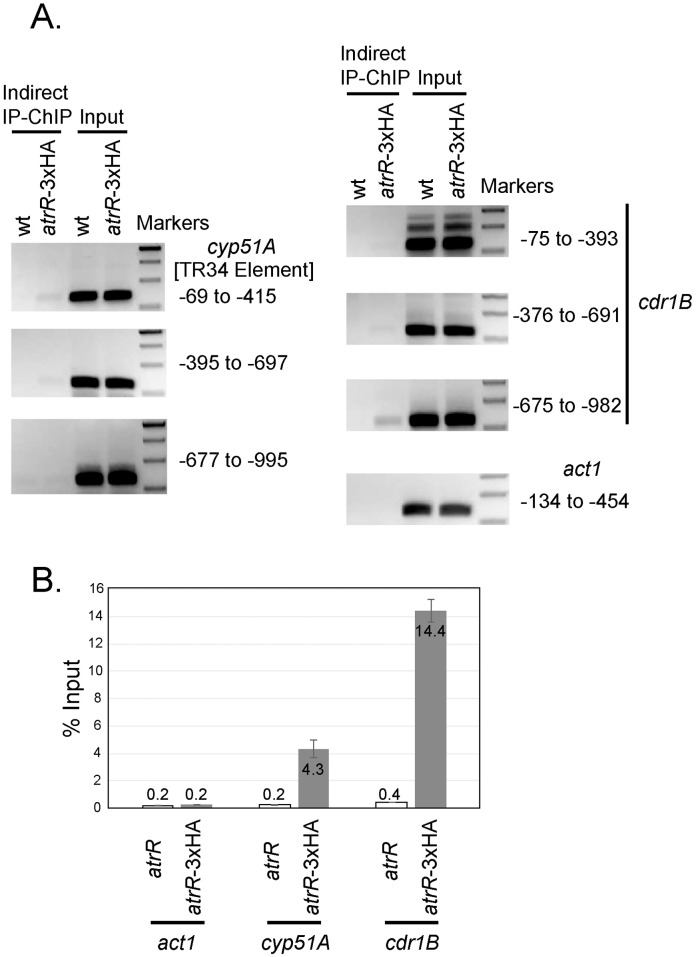
Chromatin immunoprecipitation assay showing binding of AtrR protein to the promoter regions of *cyp51A* and *cdr1B*. (A) Chromatin immunoprecipitation (ChIP) was done using a mouse monoclonal antibody directed against the HA epitope. Crosslinked chromatin was prepared from a WT strain or an isogenic strain expressing a 3x HA-tagged form of *atrR* (shown as atrR-3× HA). Immunoprecipitated DNA was examined by semiquantitative RT PCR analysis using primers that amplified the *cyp51A* and *cdr1B* promoters. The relative enrichment of the *act1* promoter was also assessed as a control for a promoter not responsive to AtrR. (B) Quantitative analysis for enrichment of the *cyp51A* and *cdr1B* promoters by real-time RT PCR method. Chromatin immunoprecipitated from the atrR-3× HA strains exhibited a 20-fold enrichment of *cyp51A* promoter DNA along with a ~33-fold enrichment of *cdr1B* promoter sequence. No detectable enrichment of *act1* was found. Data are presented as the mean and standard error of two separate ChIP experiments. % input represent signals obtained from the ChIP that are divided by signals obtained from the input sample.

To examine the mode of AtrR interaction with the promoters of *cyp51A* and *cdr1B*, we carried out chromatin immunoprecipitation (ChIP) analysis using an epitope-tagged allele of *atrR*. A 3x hemagglutinin (HA) tag was placed at the carboxy-terminus of the chromosomal *atrR* gene (S5A Fig). This allele was still able to confer azole resistance indicating that AtrR function was maintained (S5B Fig). ChIP experiments were carried out as described [30] with immunoprecipitated chromatin examined for enrichment of fragments corresponding to the promoter regions of *cyp51A*, *cdr1B* and *act1*. We found the strongest level of enrichment for the *cdr1B* promoter with easily detectable recovery of the *cyp51A* promoter (Fig 9A). ChIP experiments using the *act1* promoter did not produce any enrichment above background when comparing ChIP reactions carried out on strains lacking the HA tag versus a strain expressing the tagged AtrR protein. This specific enrichment for the *cdr1B* and *cyp51A* promoters was further confirmed by quantitative real-time PCR (Fig 9B). These data are consistent with AtrR acting at sites upstream from both *cdr1B* and *cyp51A*. Specificity of this interaction is supported by the lack of enrichment of the *act1* promoter region.

### The role of AtrR, SrbA, Cdr1B, and Cyp51A in sensitivity to azoles antifungals

To compare the physiological importance of the *atrR*, *srbA*, *cdr1B*, and *cyp51A* genes, we generated a series of isogenic strains lacking these important drug resistance loci in the Afs35 background. Representative isolates were then assessed for their drug resistance by determining their minimum inhibitory concentration (MIC) for a variety of antifungal drugs. The MICs of fluconazole, itraconazole, voriconazole, and miconazole were lowered in *ΔatrR* and *ΔsrbA* compared to the WT (Afs35) (Table 3). The MICs of voriconazole and miconazole were more depressed in the *ΔatrR* strain than in the corresponding *ΔsrbA* mutant, which are consistent with the results of growth inhibition assay using the Af293 background strains (Fig 2B). The double deletion mutant (*ΔatrR ΔsrbA*) exhibited increased susceptibility for azoles compared to either of single mutants. In the *Δcdr1B* and *Δcyp51A*, the MICs of itraconazole, voriconazole, and miconazole were lowered (50%-87.5%), compared to the WT. These drug susceptibility tests confirmed that the transcription factors AtrR and SrbA, the ABC transporter Cdr1B, and the target enzyme Cyp51A were required for normal azole resistance. Importantly, simultaneous loss of the transcription factors AtrR and SrbA caused the most profound azole sensitivity of all the genetic alterations tested.

**Table 3:** Minimal inhibitory concentrations of antifungals in *A*. *fumigatus* mutant strains.

	MEC (mg/L)*^1^		MIC (mg/L)*^1^						
Strain	MCFG	CPFG	AMPH	5-FC	FLCZ	ITCZ	VCZ	MCZ	PCZ
Afs35 (WT)	<0.015	n.d.	1	>64	>64	0.5	0.5	2	n.d.
*Δcdr1B*	<0.015	n.d.	1	>64	>64	0.25	0.125	1	n.d.
*Δcyp51A*	<0.015	n.d.	1	>64	16	0.25	0.25	0.25	n.d.
*ΔatrR*	<0.015	n.d.	1	16	4	0.125	<0.015	<0.03	n.d.
*ΔsrbA*	<0.015	n.d.	1	>64	2	0.125	0.03	0.06	n.d.
*ΔatrR*,*srbA*	<0.015	n.d.	1	>64	0.5	0.06	<0.015	<0.03	n.d.
IFM 61567	<0.015	0.25	2	64	>64	>8	1	0.5	8
IFM 61567 *ΔatrR* No.2	<0.015	0.25	2	>64	4	0.125	0.06	0.06	0.06
IFM 61567 *ΔatrR* No.3	<0.015	0.25	2	64	4	0.125	0.06	0.06	≦0.06
IFM 61567 *ΔatrR* No.4	<0.015	0.25	2	64	4	0.25	0.06	0.06	≦0.06

*^1^ n.d. indicates 'not determined'.

Micafungin: MCFG; caspofungin: CPFG; amphotericin B: AMPH; flucytosine: 5-FC; fluconazole: FLCZ; itraconazole: ITCZ; voriconazole: VRCZ; miconazole: MCZ; posaconazole: PCZ.

### Deleting *atrR* cures azole resistant phenotype in the clinical strain with Cyp51A G54E mutation

**Fig 10:**
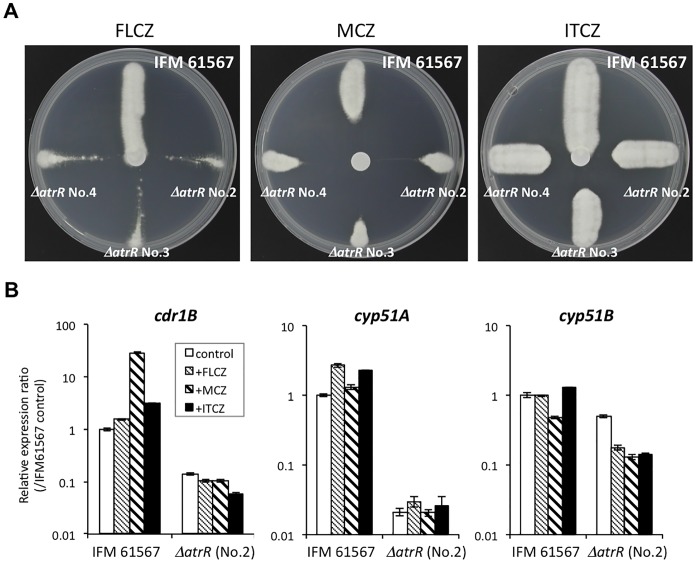
The clinical azole resistant strain with an *atrR* gene deleted shows azole hyper-susceptibility. (A) Drug susceptibility test by paper-disc diffusion assay. A paper-disc was placed on center of the GMM plate, and 10 μl of drug solution indicated was dropped on it (FLCZ: 10 mg/mL; MCZ: 10 mg/mL; ITCZ: 10 mg/mL). An azole resistant strain IFM 61567 and the independently obtained *atrR* deleted strains (*ΔatrR* No. 2–4) were streaked outward from the center. The plates were incubated for 48 h before photographed. The IFM 61567 fully grew along the streaked line regardless of the presence of FLCZ and ITCZ, whereas the *ΔatrR* mutants showed growth inhibition. (B) Expression levels of *cdr1B*, *cyp51A*, and *cyp51B* genes in IFM 61567 and the *atrR*-deleted strain (*ΔatrR* No.2) were determined by real-time RT PCR method. IFM 61567 and the *ΔatrR* were cultivated in YPG for 18 h followed by 1 h culture with or without FLCZ, MCZ, and ITCZ (final concentrations, 2 μg/mL). The expression levels of genes of interest were normalized with that of actin gene. Relative expression ratios against IFM 61567 without drug (control) are shown. Error bars represent the standard deviations based on three independent replicates.

Considering the important role of AtrR in azole resistance, we wanted to evaluate the contribution of the *atrR* gene to azole resistance in a clinical isolate. We deleted the *atrR* gene from the genome of the clinical isolate IFM 61567 that contains a known itraconazole resistance-conferring mutation in its *cyp51A* gene (Cyp51A G54E). We obtained three independent deletion mutant strains (IFM 61567 *ΔatrR* No.2-4) and compared the MICs of antifungals with the starting strain (Table 3). The parental strain IFM 61567 showed resistance to itraconazole and posaconazole, but retained susceptibility to voriconazole and miconazole. All the three *ΔatrR* deletant strains were highly susceptible to itraconazole (MIC: 0.125~0.25) and posaconazole (MIC: ≤0.06~0.06), as well as to voriconazole (MIC: 0.06) and miconazole (MIC: 0.06), which revealed these strains are not resistant to azoles, even if judged from a clinical endpoint. We also used a disc-diffusion assay to confirm azole susceptibility for these mutants upon fluconazole, miconazole, and itraconazole treatment (Fig 10A). Together, these different assays demonstrated that the *ΔatrR* strains were more sensitive to these azoles compared to the parental resistant strain (IFM 61567). We confirmed that the expression levels of *cdr1B*, *cyp51A*, and *cyp51B* were markedly decreased in the *ΔatrR* derivatives (Fig 10B), as was observed earlier for *ΔatrR* in Af293 background. Collectively, our results showed that the *atrR* gene deletion led to azole sensitization even in this clinically isolated multi-azole resistant strain with a G54E mutation in Cyp51A.

## Discussion

In the present study, we identified a novel transcription factor called AtrR that is essential for azole resistance in the human pathogen *A*. *fumigatus* as well as the industrially important fungus *A*. *oryzae* and the model fungus *A*. *nidulans*. Our results using the ChIP assay support the view that AtrR exerts direct transcriptional regulation on *cyp51A* and *cdr1B* expression via promoter association. In filamentous fungal pathogens, AtrR is the second transcriptional regulator, after SrbA, regulating the *cyp51A* gene. To the best of our knowledge, this is the first report of a direct transcriptional regulator for azole resistance-related ABC transporter genes in filamentous fungi including *A*. *fumigatus*. AtrR is widely found in an array of filamentous fungi including plant and human pathogenic fungi. Therefore, this finding sheds important new light on how filamentous fungi react and adapt to azole challenge via the coordinate upregulation of both sterol biosynthesis and ABC transporter gene expression.

### Regulation of ABC transporters in *A*. *fumigatus*

The ABC transporters in the PDR sub-family are one of the main players implicated in azole resistance in fungi [34]. It has been demonstrated in many fungi, including plant and human pathogens, that certain ABC transporter genes were up-regulated in response to azole treatment, and loss of genes encoding PDR-type ABC transporters resulted in hypersensitivity to azoles [35–37]. According to phylogenetic analysis, the *A*. *fumigatus* PDR sub-family (also called ABCG family) contains 12 ABC transporters [38]. Among these include the already characterized Cdr1B, AbcA, AtrF, and AtrI, deletion mutants of which showed decreased susceptibility to azoles [20,22,39,40]. Our RNA-seq analysis showed that only a few ABC transporter genes were upregulated upon miconazole and fluconazole treatment, and that Cdr1B seemed to be the only transporter regulated by AtrR (Table 2). Studies of Cdr1B revealed a clinical relevance as an azole resistant strain overexpressing *cdr1B* has been isolated from a patient [20]. In that report, genetic evidence was provided that azole resistance was caused by *cdr1B* overexpression. However, the mechanism of that overexpression was not assessed in the paper. It is possible that elevated activation via AtrR led to the increased *cdr1B* transcription.

In a model filamentous fungus *Neurospora crassa*, Cdr4, an ortholog of Cdr1B or *S*. *cerevisiae* Pdr5p, plays a major role in azole tolerance [41]. Although direct interaction with the promoter region was not investigated, two transcription factors were characterized to be involved in regulating the *cdr4* expression. The putative transcription factor CCG-8, which has been identified as a clock-controlled gene, positively regulates *cdr4* as well as the azole target gene *erg11*, an ortholog of *A*. *fumigatus cyp51A* [42]. Ketoconazole challenge induced *cdr4* and *erg11* expression in the WT, whereas the induction levels were partly decreased in the *ccg-8* deletion mutant. The CCG-8 ortholog (Afu5g09420) in *A*. *fumigatus* has not been characterized so far. A role of the bZip transcription factor ADS-4 was also investigated with regard to azole resistance in *N*. *crassa* [43]. A null mutant lacking *ads-4* showed hypersensitivity to itraconazole and ketoconazole. ADS-4 was involved in regulating *cdr4* as well as *erg5* encoding a sterol C-22 desaturase. The ortholog of *ads-4* (Afu1g16460) was also characterized in *A*. *fumigatus* with its deletion strain, which showed a slightly higher sensitivity to azoles compared to the parental strain. Our RNA-seq data revealed that expression of the two transcription factors (CCG-8 and ADS-4) was not affected by *atrR* deletion in *A*. *fumigatus* (Table 2), which supports the idea that AtrR is an independent regulator of *cdr1B* and *cyp51A* expression. The molecular mechanism of how AtrR functions will be an essential goal of future study.

In the pathogenic yeasts, *C*. *albicans* and *C*. *glabrata*, the molecular mechanisms underlying transcriptional regulation of ABC transporters (functioning as drug efflux pump) have been more intensively studied. The azole resistance-related ABC transporter Cdr1 (an ortholog of *A*. *fumigatus* Cdr1B and *S*. *cerevisiae* Pdr5p) is directly regulated by a Zn_2_-Cys_6_ transcription factor Pdr1 in *C*. *glabrata* [44]. In a clinical setting, azole resistant strains with elevated *CDR1* expression were commonly recovered. In most of the strains, *CDR1* overexpression was attributed to constitutively activated Pdr1 that possessed a gain-of-function mutation [45,46]. As stated earlier, *Candida* has no ortholog of AtrR. Indeed, *Candida* Pdr1 and *A*. *fumigatus* AtrR only share sequence homology in a Zn_2_-Cys_6_ domain at the N-terminus. The most notable distinctive role between the ABC transporter regulating transcription factors, Pdr1 and AtrR, is that AtrR expression is not substantially upregulated in response to azole treatment (Fig 7B), whereas Pdr1 itself is induced by azole treatment [47]. Collectively our results suggest that the transcriptional regulator of azole resistance-related ABC transporters, particularly for a Cdr1-type transporter, is evolutionarily distinct between yeasts and filamentous fungi.

### Regulation of *cyp51A* gene by AtrR in association with SrbA

Ergosterol biosynthesis is a crucial process that is specific to fungi. Antifungal drugs such as azoles and polyenes target this biosynthetic pathway or the ergosterol molecule directly. The SREBP SrbA is a well-studied transcription factor that regulates ergosterol pathway-related genes including *erg3*, *erg24*, *erg25*, and *cyp51A* [27,30]. Deletion of *srbA* resulted in a disturbed sterol profile and hypersusceptibility to azole drugs. Intriguingly, a deletion mutant of *atrR* revealed strikingly similar phenotypes to those of the *srbA* deletion mutant. In addition to the regulation of *erg* genes and azole susceptibility, hypoxia adaptation and virulence attenuation were phenocopied between the *srbA* and *atrR* null mutants. This striking overlap argues that AtrR and SrbA closely function to regulate the ergosterol biosynthesis pathway. Recent studies provided information on the contributors in the SREBP pathway including the Golgi Dsc E3 ligase complex members (DscA-E), a rhomboid family protease RbdB (also reported as RbdA), and an aspartyl peptidase SppA [48–51]. Although these contributors are essential for SrbA function, transcription levels of these genes including *srbA* were not largely affected by deleting AtrR (Table 2). This suggested that AtrR did not act upstream via the SREBP pathway at a transcriptional level but rather may have its own independent regulatory input. The possibility that AtrR interacts with SrbA to function as a transcriptional regulator complex over the genes related to ergosterol biosynthesis, thus far, cannot be ruled out.

Previous studies by others revealed that SrbA binds to the promoter region of the *cyp51A* gene (also known as *erg11A*) [27,30]. ChIP-seq data indicated that SrbA-enrichment peaks resided in the *cyp51A* promoter region at the site 302 bp upstream from the initiation codon [30]. Accordingly, a putative SrbA binding site similar to the mammal SRE binding motif (TCACNCCAC) was found in this same region. Notably, the peak of SrbA-enrichment and the putative binding site are contained within a 34 bp sequence that had been identified as a tandem repeat sequence in azole resistant strains [11,13]. In our ChIP data for *cyp51A* with AtrR-3HA, a slightly more intense band was exhibited by probing with -69 to -415 region than with -395 to -697 (Fig 9A). This suggested that the binding motif of AtrR in the *cyp51A* promoter is more likely to be present at the region from -69 to -415 rather than -395 to -697. Gal4-type Zn_2_-Cys_6_ transcription factors typically bind to inverted CGG triplets as homodimers [52]. We therefore searched for CGG-N_x_-CCG motifs in the *cyp51A* promoter region (to -1000). Interestingly, we found three regions with the motifs, CGG-N_10_-CCG, CGG-N_18(9)_-CCG, CGG-N_17(12)_-CCG, at -949 to -934, -796(-787) to -773, and -314(-309) to -292, respectively. The last one is located in the 34 bp of tandem repeat-related sequence and thus is adjacent to the putative SrbA-binding site. From these data, we anticipate that AtrR and SrbA could cooperatively function to regulate *cyp51A* expression at the proximal sites in the promoter region. Additionally, three motifs (CGG-N_5_-CCG, CGG-N_21_-CCG, and CGG-N_20_-CCG) were found in the *cdr1B* promoter at the sites (-723 to -713, -537 to -511, and -495 to -470). For the other 51 AtrR-dependent genes identified in RNA-sequencing analysis, the potential CGG-N_x_-CCG motifs were widely detected in the promoter region (-1000 to 0) of several genes (S1 Table). Determination of the accurate binding sequences for AtrR is crucial to allow detailed understanding of the molecular function of AtrR.

### Physiological impact of AtrR on hypoxia adaptation and pathogenicity

As molecular oxygen is a critical component for several biochemical processes, fungi must have the ability to adapt to hypoxic conditions. Previous studies showed that ergosterol biosynthesis is one of the fungal metabolic pathways most responsive to oxygen level [27,32,53,54]. Several enzymes in the fungal ergosterol biosynthesis pathway, such as the cytochrome P450 Erg3 and Erg11/Cyp51, require molecular oxygen as well as heme. Heme biosynthesis is also an oxygen-requiring pathway. The SREBP pathway coordinates oxygen levels with sterol and heme biosynthesis since these pathways are responsive to levels of this gas [28,30,55]. In the present study, AtrR was found to be involved in hypoxia adaptation as seen before for SrbA. In fact, the expression levels of *cyp51A*, *erg3B*, *erg24A*, and *erg25A* were induced in response to oxygen limitation in an AtrR- and SrbA-dependent manner (Fig 5B). Although the mechanism is still unknown, AtrR is responsible for sterol regulation under oxygen-limited condition, likely in association with SREBP pathway.

Sterol regulation of *S*. *cerevisiae* and *Candida* species involves a Gal4-type Zn_2_-Cys_6_ transcription factor Upc2. Interestingly, these yeasts lack an SREBP pathway. A recent finding in the lipid degrading yeast *Yarrowia lipolytica* that retains an intact SREBP, called YlSre1, revealed that YlSre1 only played a minor role while Upc2 played a major role in sterol regulation, hypoxia adaptation and azole resistance [56]. Therefore it was proposed that Upc2 has replaced the role of SREBPs in sterol regulation in these yeasts. From a comparison of the amino acid sequences, it is evident that AtrR is not an orthologue of Upc2.

Several lines of data have supported an intimate relationship between hypoxic adaptation and pathogenicity of pathogenic fungi. 1) The murine lung infected by fungi is a hypoxic environment at the infection site, which was revealed by a hypoxic indicator [57], and 2) SREBP mutants of *C*. *neoformans* (Sre1) and *A*. *fumigatus* (SrbA) which are unable to grow under hypoxic condition showed an avirulent phenotype [27,32]. We demonstrated that AtrR is also required for both hypoxic adaptation and *A*. *fumigatus* virulence. As AtrR is a fungal specific transcription factor, it would be a potential drug target, inactivation of which could suppress *A*. *fumigatus in vivo* growth during the infection process.

### Deleting AtrR overcame Cyp51A-related azole resistance

One of the most noteworthy results in the present study is that deleting the *atrR* gene effectively sensitized the clinically isolated multi azole-resistant strain (Fig 10A and Table 3). Loss of *atrR* from this clinical strain reduced expression levels of *cyp51A* and *cyp51B* to the low level seen in the other laboratory lineage (Af293-background) (Fig 10B). This, in turn, likely reduced the amount of target proteins, Cyp51A and Cyp51B, in the cells. Even though the strain carried a G54E substitution in the Cyp51A protein, the reduction in gene transcription contributed to the resulting strains showing hypersensitivity to azoles in contrast to the resistant parental strain IFM 61567. Alternatively, accumulation of toxic intermediates at multiple AtrR-regulated steps (Erg3B, Cyp51A, Erg24A, and Erg25A) in the ergosterol biosynthesis pathway could also contribute to reacquisition of azole sensitivity. Even when *cyp51A* expression was heterologously elevated in an *atrR* background, this was insufficient to suppress the azole-susceptibility of this mutant strain (Fig 3B). The same result was also observed in *srbA* mutant background (Fig 3B), and is consistent with earlier work [29]. These results suggested that defective *cyp51A* expression was not the only reason for the azole-sensitivity in *atrR* or *srbA* deletion mutants. If judged by MIC values caused by loss of AtrR (itraconazole: 0.125–0.25; voriconazole: 0.06; posaconazole: ≦0.06–0.06), the resultant deletion mutants would be curable in a conventional azole drug therapy. This fact clearly illuminates a path toward development of new azole-sensitizing agent that could overcome Cyp51A-related azole resistance mechanisms.

In this study, we characterized a transcriptional factor designated AtrR that is responsible for a novel *A*. *fumigatus* intrinsic azole resistance mechanism. AtrR crucially governs adaptation to azole treatment in the sense that both target (*cyp51A*) and efflux pump (*cdr1B*) are transcriptionally co-regulated by AtrR. The experiment using a clinical azole-resistant isolate revealed a potential clinical relevance of AtrR. Our findings provide the first direct evidence that AtrR serves as a critical coordinator of both ergosterol biosynthesis and levels of a plasma membrane ABC transporter that can efflux azole drugs, the major antifungal drug in current use against pathogenic fungi.

## Materials and Methods

### Ethics statement

The principles that guide our studies are based on the Guidelines for Proper Conduct of Animal Experiments formulated by Science Council of Japan in June 1, 2006. All protocol used in this study were approved by the institutional animal care and use committee of Chiba University (Permit Number: DOU25-207, DOU26-228, and DOU28-345). All efforts were made to minimize suffering in strict accordance with the principles outlined by the Guidelines for Proper Conduct of Animal Experiments. Animals were clinically monitored at least daily and humanely sacrificed if moribund (defined by lethargy, dyspnea and weight loss). All stressed animals were sacrificed by cervical dislocation.

### Strains and growth media

*A*. *oryzae ΔligD*::*sC* (*niaD*^*−*^) [58] and *A*. *nidulans Δku70* (*pyrG*^*−*^, *pyroA*^*−*^, *biA1*^*−*^, *ku70*::*argB*) strains (derived from the strains, RIB40 and ABPU1, respectively) were used for a generation of deletion mutants of the AtrR-family genes. For identification of the *AoatrR*, the *A*. *oryzae* strains overexpressing a TF gene were used, which were obtained from a genetic library prepared by Noda Institute for Scientific Research, Kikkoman Corporation [33]. Briefly, the transcription factor genes of interest were cloned into an overexpression vector (pAPLTN) [59], which drives the expression under the *amyB* promoter system in the presence of maltose as a sole carbon source. *A*. *fumigatus* strains Af293 and Afs35 (*akuA*::*loxP*) were used to generate the following deletion strains and *atrR* complemented strain: *ΔatrR*, *Co-atrR*, *ΔsrbA*, *Δcyp51A*, *Δcdr1B*, *ΔatrR*,*srbA*, Af293+P*thiA*-*cyp51A*, *ΔatrR*+P*thiA*-*cyp51A*, and *ΔsrbA*+P*thiA*-*cyp51A*. The clinical strain IFM 61567 was isolated in 2011 in Japan from a patient with itraconazole treatment. The mutation in *cyp51A* gene in the strain was identified as a procedure described previously [60]. The fungal strains used in this study are listed in Table 4. All strains were routinely cultivated in potato dextrose agar (PDA), potato dextrose broth (PDB), 0.1% yeast extract-containing glucose minimal medium (YGMM), or glucose minimal medium (GMM) at 37°C. For *A*. *oryzae* or *A*. *nidulans* culture, Czapek-Dox (CD) medium was used, which contained 1% maltose or 1% glucose, 68.8 mM (NH_4_)_2_SO_4_, trace elements (1 μg/ml FeSO_4_·7H_2_O, 8.8 μg/ml ZnSO_4_·7H_2_O, 0.4 μg/ml CuSO_4_·5H_2_O, 0.15 μg/ml MnSO_4_·4H_2_O, 0.1 μg/ml Na_2_B_4_O_7_·10H_2_O, 0.05 μg/ml (NH_4_)_6_Mo_7_O_24_·4H_2_O) and appropriate supplements such as 1 mg/ml uridine, 0.02 μg/ml biotin, 2.5 μg/ml pyridoxine. To collect conidia of each *A*. *fumigatus* strain, PDA was used. The antifungal chemicals were commercially obtained as follows: fluconazole, miconazole, itraconazole, propiconazole, bromuconazole, tebuconazole, difenoconazole, clotrimazole (Wako Pure Chemical Industries, Osaka, Japan), and amphotericin B (Sigma-Aldrich Co., St. Louis, MO, USA). Micafungin was a generous gift from Dr. Keietsu Abe, Tohoku University.

**Table 4:** Strains used in this study.

Strain	Background strain	Genotype
*A*. *oryzae* RIB40 niaD300	WT	*niaD-*
*A*. *oryzae*+pAPLTN	RIB40	*niaD+*
*A*. *oryzae*+TF1	RIB40	*niaD+*, P*amyB*-TF1
*A*. *oryzae*+TF2	RIB40	*niaD+*, P*amyB*-TF2
*A*. *oryzae*+TF3	RIB40	*niaD+*, P*amyB*-TF3
*A*. *oryzae*+TF4	RIB40	*niaD+*, P*amyB*-TF4
*A*. *oryzae*+TF5	RIB40	*niaD+*, P*amyB*-TF5
*A*. *oryzae* LigD	RIB40	*ΔligD*::*sC*, *niaD-*
*A*. *oryzae ΔatrR*	RIB40	*ΔligD*::*sC*, *niaD-*, *AoatrR*::*ptrA*
*A*. *nidulans* KU70	ABPU1	*pyrG-*, *pyroA-*, *biA1-*, *ku70*::*argB*
*A*. *nidulans ΔatrR*	KU70	*pyrG-*, *pyroA-*, *biA1-*, *ku70*::*argB*, *AnatrR*::*ptrA*
Af293	WT	-
*ΔatrR*	Af293	*atrR*::*hph*
*Co-atrR*	Af293	*atrR*::*hph*, *atrR*^*+*^*-ptrA*
*ΔsrbA*	Af293	*srbA*::*ptrA*
Af293+*PthiA-cyp51A*	Af293	*PthiA-cyp51A-phle*
*ΔatrR+PthiA-cyp51A*	Af293	*atrR*::*hph*, *PthiA-cyp51A-phle*
*ΔsrbA+PthiA-cyp51A*	Af293	*srbA*::*ptrA*, *PthiA-cyp51A-phle*
AfS35	WT	*ΔakuA-loxP*
*ΔatrR*	AfS35	*ΔakuA-loxP*, *atrR*::*hph*
*ΔsrbA*	AfS35	*ΔakuA-loxP*, *srbA*::*ptrA*
*ΔatrR*,*srbA*	AfS35	*ΔakuA-loxP*, *atrR*::*hph*, *srbA*::*ptrA*
*Δcdr1B*	AfS35	*ΔakuA-loxP*, *cdr1B*::*ptrA*
*Δcyp51A*	AfS35	*ΔakuA-loxP*, *cyp51A*::*ptrA*
SPF89	AfS35	*ΔakuA-loxP*, *atrR*-3xHA-*hph*
IFM 61567	-	*cyp51A*(G54E)
IFM 61567 *ΔatrR* No2	IFM 61567	*cyp51A*(G54E), *atrR*::*hph*
IFM 61567 *ΔatrR* No3	IFM 61567	*cyp51A*(G54E), *atrR*::*hph*
IFM 61567 *ΔatrR* No4	IFM 61567	*cyp51A*(G54E), *atrR*::*hph*

### Construction of the gene deletion and complemented strains

For the deletion of the *AoatrR* gene in *A*. *oryzae*, approximately 1-kb fragments upstream and downstream of the coding region of the gene were amplified by PCR using primers YESAoAtrRFw + AoAtrRPtrARv and PtrAAoAtrRFw + AoAtrRYESRv, respectively. The selectable marker gene, *ptrA*, was isolated by digesting pPTRI (TaKaRa Bio, Otsu, Japan) with *Nhe*I. Then, the PCR-amplified two DNA fragments, the *ptrA* gene, and a yeast vector, pYES2 (Invitrogen, Tokyo, Japan), digested with *Eco*RI and *Bam*HI, were assembled in *S*. *cerevisiae* BY4741 (*MAT*a, *his3Δ1*, *leu2Δ0*, *met15Δ0*, *ura3Δ0*) as described previously [61,62]. The resulting plasmid DNA was digested with *Kpn*I and *Not*I to prepare a deletion construct, which was used for deletion of *AoatrR*. Similarly, for the deletion of the *AnatrR* gene in *A*. *nidulans*, DNA fragments upstream and downstream of the coding region of the gene were PCR-amplified using primers YESAnAtrRFw + AnAtrRPtrARv and PtrAAnAtrRFw + AnAtrRYESRv, respectively. The PCR-amplified two DNA fragments, the *ptrA* gene, and pYES2 digested with *Eco*RI and *Bam*HI were assembled in *S*. *cerevisiae*, and used for deletion of *AnatrR*. Southern blot analyses demonstrated that the transformation cassettes had integrated homologously at the targeted loci and the target ORF was replaced with a selectable marker gene. To construct the *A*. *fumigatus* deletion mutants and the complemented strain, plasmids for each purpose were generated. DNA manipulation was performed according to standard laboratory procedures. To amplify DNA fragments from the genome, Prime STAR HS (TaKaRa Bio) was used. To prepare gene replacement cassettes for *A*. *fumigatus*, the fragments and plasmids were constructed by one-step fusion PCR and GeneArt Seamless Cloning and Assembly Kit (Invitrogen), respectively. Primers used in the present study were listed in S3 Table.

To generate a replacement cassette for *atrR* gene, the 5′- and 3′-flanking regions were obtained using the primers AtrR-5.UP and AtrR-5.LP(hph) (for the 5′-region) and AtrR-3.UP(hph) and AtrR-3.LP (for the 3′-region). These flanking regions and an hph fragment, hygromycin B resistant marker that was amplified from pCB1004 plasmid, were fused by one-step fusion PCR, which were then used for transformation.

For complementation of *atrR* gene, the *atrR* fragment containing both upstream and downstream sequences were PCR amplified using the primers AtrR-5.UPHind and AtrR-3.LP. The PCR fragment was digested by *Hin*dIII and *Xma*I and ligated into *Hin*dIII- and *Hpa*I-digested pKIS518 (derived from a binary vector pPZP-HYG2, in which the *hygB* gene was removed by *Bam*HI digestion, and a *ptrA* cassette is ligated at *Kpn*I and *Eco*RV cites between the right and left borders of T-DNA), resulting in a plasmid pKIS518-atrR, which was used for *Agrobacterium tumefaciens*-mediated transformation.

To generate replacement cassettes for the *srbA*, *cyp51A*, and *cdr1B* genes, the 5′- and 3′-flanking regions were obtained using the primers srbA-U-F(pUC119E) and srbA-U-R(ptrA) (for the 5′-region of *srbA*) and srbA-D-F(ptrA) and srbA-D-R(pUC119B) (for the 3′-region of *srbA*), cyp51A-U-F(pBC-ph-s) and cyp51A-U-R(ptrA) (for the 5′-region of *cyp51A*) and cyp51A-D-F(ptrA) and cyp51A-D-R(pBC-ph-x) (for the 3′-region of *cyp51A*), and cdr1B-U-F(pUC119B) and cdr1B-U-R(ptrA) (for the 5′-region of *cdr1B*) and cdr1B-D-F(ptrA) and cdr1B-D-R(pUC119E) (for the 3′-region of *cdr1B*), respectively. These flanking regions and a *ptrA* fragment, pyrithiamine resistant marker that was amplified from pPTRI plasmid (TaKaRa Bio), were fused into pUC119 or pBC-phleo by GeneArt cloning system, resulting in plasmids pUC119-srbA::ptrA, pBC-phleo-cyp51A::ptrA, and pBC119-cdr1B::ptrA. The cassettes used for transformation were amplified from these plasmids.

For generation of a plasmid pBC-phleo-PthiA::cyp51A, promoter fragment (approximately 800bp) of *A*. *fumigatus thiA* (Afu6g08360) and the *cyp51A* ORF and terminator (approximately 450bp) fragment were obtained using primers PthiA-F(pBC-ph-s) and PthiA-R(cyp51A) (for the PthiA) and RT-cyp51A-F and cyp51A+T-R(pBC-ph-x) (for the *cyp51A* ORF+ter), respectively. These fragments were fused into pBC-phleo by GeneArt cloning system, resulting in a plasmid pBC-phleo-PthiA::cyp51A, which was used for transformation to obtain strains, Af293+P*thiA*-*cyp51A*, *ΔatrR*+P*thiA*-*cyp51A*, and *ΔsrbA*+P*thiA*-*cyp51A*.

*A*. *fumigatus* transformation was performed according to a protoplast-polyethylene glycol transformation method for *Aspergillus* [63]. Precise recombination and integration were confirmed by Southern Blot analysis and/or PCR of the genomic DNA, and in case of gene deletion the absence of mRNA of the target gene was confirmatively verified using real-time RT-PCR analysis. To generate a complemented strain for the *atrR* gene, *A*. *tumefaciens* strain EHA105 carrying the pKIS518-atrR was used for transformation of *A*. *fumigatus ΔatrR* strain by the method described elsewhere [64].

### Antifungal drugs susceptibility test

Paper-disc diffusion assay: The conidia of each strain of interest were mixed with 20 mL of GMM (final concentration, 10^4^ conidia/mL), and they were pored into a petri dish to solidify. A paper-disc was placed on center of the plate, and 10 μl of drug solution was dropped on it (fluconazole: 10 mg/mL; miconazole: 10 mg/mL; amphotericin B: 0.25 mg/mL; micafungin: 0.02 mg/mL). The plates were incubated at 37°C for 48 h before photographed. Instead of mixing conidia with pre-solidified GMM, the strains were inoculated by streaking conidia on a GMM plate when multiple strains were investigated.

Colony growth inhibition test: The 10^4^ conidia of each strain were inoculated on the center of GMM plates containing 0 to 5 μg/mL of itraconazole or miconazole. The plates were incubated at 37°C for 70 h. The colony diameter was measured from three independent plates, and the mean values for plates with drugs were compared with those without drugs.

MIC test: MICs of each strain against antifungal drugs were investigated as described previously [65]. Tests were performed in triplicates using micafungin, amphotericin B, flucytosine, fluconazole, itraconazole, voriconazole, miconazole, and posaconazole in RPMI 1640 medium (pH 7.0) at 35°C. This was performed according to the Clinical and Laboratory Standards Institute reference broth microdilution method, document M38-A2, with partial modifications in term of using the dried plate for antifungal susceptibility testing of yeasts method (Eiken Chemicals, Tokyo, Japan).

### RNA and cDNA preparation

The mycelia were cultured in GMM, YGMM, or PDB at 37°C and harvested at time-points (18~24 h) appropriate for the applications. The mycelia were frozen in liquid nitrogen, and total RNA was isolated using the FastRNA Pro Red Kit (MP Biomedicals, Santa Ana, CA, USA). To obtain cDNA pools from the total RNA, reverse transcription was performed using the ReverTra Ace qPCR RT Master Mix with gDNA remover (Toyobo, Osaka, Japan).

### Quantitative real-time RT-PCR

Real-time RT-PCR was performed using SYBR Green detection as described previously [63]. The Thunderbird SYBR qPCR Mix was used for reaction mixture preparation (Toyobo). The primer sets are listed in S3 Table. The relative expression ratios were calculated by the comparative cycle threshold (Ct) (ΔΔCt) method. The actin gene was used as a normalization reference (internal control). Each sample was tested in triplicate.

### RNA-sequencing analysis

RNA-sequencing analysis was performed as described previously [66]. Briefly, mRNA libraries and cDNA libraries were prepared by Illumina TruSeq RNA Sample Prep Kit v2 according to the standard protocols (Illumina, San Diego, CA, USA). Each total RNA sample (1 μg) was enriched for mRNA using oligo (dT)-tagged beads. The library construction involved cDNA synthesis, end repair, A-tailing, adapter ligation, and amplification. The mean length for each library was approximately 280 to 300 bp. Sequencing was performed in a pair-end 50 base mode on a Miseq system (Illumina). In this study, we performed two independent experimental settings: one containing Af293, *ΔatrR*, and *ΔsrbA* (total 3 samples) and the other containing Af293 and *ΔatrR* with DMSO, fluconazole, or miconazole treatment (total 6 samples). These read data were deposited to the DDBJ Sequence Read Archive under accession No. PRJDB5273.

To compare expression levels for each gene, the sequences were analyzed using CLC genomics workbench (CLC Bio, Aarhus, Denmark). The sequence reads were trimmed on the program software. The only reads with quality values higher than Q30 were used for mapping. The *A*. *fumigatus* Af293 genome data retrieved from NCBI (http://www.ncbi.nlm.nih.gov/bioproject/PRJNA14003) was used as the template for mapping. From the mapping data, RPKM values were calculated. Heat map presentation was performed using Cluster 3.0 and Java TreeView ver 1.1.6r2 according to the instructions.

### Growth under hypoxia condition

The conidia of each strain were inoculated on GMM plate, and the plate was incubated at 37°C in anaeropack system (Mitsubishi Gas Chemical, Tokyo, Japan) where initial concentration of oxygen was set at 2% (hypoxia condition). After 96 h, oxygen in the pack appeared to be completely consumed as estimated from no more growth of all colonies on the plate. The plate for normoxia was incubated at 37°C in a regular incubator without gas control (21% oxygen) for 72 h.

### Murine model of pulmonary aspergillosis

The mouse model of pulmonary aspergillosis was performed according to [67] with slight modifications. *A*. *fumigatus* strains Af293, *ΔatrR*, and *Co-atrR* were used to infect immunosuppressed mice (12 mice per group). Outbreed male ICR mice (5w, body weight, 20 to 24 g) were housed in sterile cages with sterile bedding and provided with sterile feed and drinking water containing 300 μg/mL tetracycline hydrochloride to prevent bacterial infection. Mice were immunosuppressed with cyclophosphamide at a concentration of 200 mg per kg of body weight, which was administered intraperitoneally on days −4, −2, 1, 3, 5, and 7 prior to and post-infection (day 0). The *A*. *fumigatus* conidia used for inoculation were grown on PDA for 5 days prior to infection. Fresh conidia were harvested in PBS+0.01% Tween 20 and filtered through a Falcon Cell Strainer (Corning Inc., NY, USA). Conidial suspensions were spun for 5 min at 3,000 × g, washed twice with PBS+0.01% Tween 20, counted using a hemocytometer, then resuspended at a concentration of 10^6^ conidia/μl. Mice were anesthetized by ketamine and xylazine and infected by intratracheal instillation of 2.5 × 10^7^ conidia in 25 μl of PBS. Mice were weighed and visually inspected every 24 h from the day of infection. The endpoint for survival experimentation was identified when a 30% reduction in body weight was recorded, at which time the mice were sacrificed. The statistical significance of comparative survival values was calculated using log rank analysis using the Prism statistical analysis package.

For *A*. *fumigatus* strains Afs35, *ΔatrR*, *ΔsrbA*, and *ΔatrR ΔsrbA*, immunosuppressed ICR mice (5w, body weight, 20 to 24 g) were used as described above (9 to 11 mice per group). Mice were immunosuppressed with cyclophosphamide at a concentration of 150 mg per kg of body weight, which was administered intraperitoneally on days −4, −1, 3, 6, 9, and 12 prior to and post-infection (day 0). Cortisone acetate (Sigma-Aldrich Co) was also administrated subcutaneously at a concentration of 200 mg per kg of body weight on day -1. Mice were anesthetized by ketamine and xylazine and infected by intratracheal instillation of 3 × 10^5^ conidia in 30 μl of PBS. Mice were weighed and visually inspected every 24 h from the day of infection. The endpoint for survival experimentation was identified when a 30% reduction in body weight was recorded, at which time the mice were sacrificed.

### Chromatin immunoprecipitation

For ChIP experiments, a strain possessing 3x HA-tagged allele of *atrR* was constructed. A cassette consisting of 750 bp of the C-terminus of *atrR* fused to a 3x HA tag, a *trpC* terminator followed by a hygromycin resistance gene and 750 bp of the 3’ untranslated region of the *atrR* gene was transformed into Afs35 cells with selection for hygromycin resistance. Resistant clones were confirmed to contain a homologous integration by PCR (Designated SPF89 strain). 1×10^6^ spores/mL of *A*. *fumigatus* strains AfS35 (*atrR*) and SPF89 (*atrR*-3xHA) were grown in 50 mL of Sabouroud dextrose medium in 250 mL shaking flask cultures for 16 h. Cross-linking was done in (0.4 M Sucrose, 10 mM Tris-HCl, pH 8.0, 1 mM EDTA, adding 1 mM PMSF and 1% formaldehyde just before use) for 15 min under shaking (100 rpm) at 30°C. Crosslinking was stopped by adding 2.5 mL of 2 M glycine, and continued shaking incubation for 10 min. Mycelia were collected and dried using vacuum filtration and rinsed with sterile ddH_2_O and frozen immediately with liquid nitrogen and stored at −80°C. Approximately 100 mg of frozen mycelia were ground to a fine powder in a chilled mortar and pestle with liquid nitrogen added. Powder was transferred to 0.5 mL of ChIP lysis buffer (CLB: 50 mM HEPES pH 7.5, 150 mM NaCl, 1 mM EDTA, 1% Triton X-100, 0.1% Deoxycholate (Sigma D6750), 0.1% SDS, 1 mM PMSF, 1× fungal proteinase inhibitor cocktail (Sigma)). Each sample was vortexed for 2 min and then split into 3*130 μl volume in AFA Fiber Pre-Slit Snap-Cap [6 x 15mm] microTUBE [Covaris] (130μl sample per tube). Chromatin was sheared with E220 Focused-ultrasonicator [Covaris] under following conditions: Peak Incident Power (W): 175; Duty Factor: 10%; Cycles per burst: 200; Treatment Time: 420 sec; Temperature: 7°C; Sample volume: 130 μl; in the presence of E220 –Intensifier (pn500141). Tubes from each sample were then pooled and centrifuged at 10,000 *g* for 5 min at 4°C. Supernatant was transferred into new tube. 25 μL was reserved as input control (IC) fraction for reverse crosslinking to verify sonication and control for ChIP and qPCR.

The sheared chromatin was incubated with HA.11(16B12) mouse monoclonal antibody (Covance) at 1:150 dilution overnight (12 h) on a nutator at 4°C. This sample was further incubated with 30 μL of washed Protein G Dynabeads (Life Technologies) for another 12 h. Washing, reverse-crosslinking and purification of ChIP-ed DNA was performed as described in Chung et al. [30].

### ChIP qRT-PCR

Semiquantitative PCR was initially performed across the *cyp51A* and *cdr1B* promoters to serve as initial verification of enrichment of these promoter regions upon ChIP with HA. This PCR was done using Phusion DNA polymerase (NEB) under following conditions: 95°C for 30 s followed by 25 cycles of 95°C for 15 s, 60°C for 10 s and 72°C for 10 s. The primers used for the reaction were cyp51A-ChIP-F1 and cyp51A-ChIP-R1, cyp51A-ChIP-F2 and cyp51A-ChIP-R2, and cyp51A-ChIP-F3 and cyp51A-ChIP-R3 (for the *cyp51A* promoter (-69 to -415, -395 to -697, and -677 to -995, respectively)), cdr1B-ChIP-F1 and cdr1B-ChIP-R1, cdr1B-ChIP-F2 and cdr1B-ChIP-R2, and cdr1B-ChIP-F3 and cdr1B-ChIP-R3 (for the *cdr1B* promoter (-75 to -393, -376 to -691, and -675 to -982, respectively)), and act1-ChIP-F and act1-ChIP-R (for the *act1* promoter (-134 to -454)) (S3 Table). Real-time PCR was performed in triplicate for each separate ChIP experiment using primers designed for regions as identified above as enriched in preliminary analysis, under the following conditions: 1 cycle of 95°C for 30 s followed by 40 cycles of 95°C for 15 s and 60°C for 30 s on an MyiQ2 BioRad machine. 1 μl of ChIP-ed or input (diluted 25-fold to bring it to 1%) DNA was used in 20 μl total volume reaction using SYBR green master mix (BioRad) and 0.4 μM of each primer. Percent input method was used to calculate the signal of enrichment of the promoter region for each gene (http://www.thermofisher.com/us/en/home/life-science/epigenetics-noncoding-rna-research/chromatin-remodeling/chromatin-immunoprecipitation-chip/chip-analysis.html).

## Supporting Information

S1 Fig Characters of AtrR-family protein. (A) Amino acid sequence alignment for Zn-finger motifs. Amino acid sequences of the candidate *A*. *oryzae* proteins (TF1 to TF5) were compared with that of *S*. *cerevisiae* Zn-finger type transcription factors, Pdr1 and Pdr3. The sequence numbers of amino acid in each protein were shown in brackets. (B) Protein motifs in the AtrR proteins. The Gal4-like Zn_2_-Cys_6_ DNA binding motif (IPR001138) is indicated by the diagonal line-containing box. The fungal specific sequence motif associated with transcription factor (IPR007219) is indicated by a dotted box. The number above the boxes show the amino acid sequence numbers counted from an initial methionine.
(PPTX)

S2 Fig Construction of *AoatrR* and *AnatrR* gene deletion strains. (A) Genomic structures of the *AoatrR* loci in *A*. *oryzae* LigD and *ΔAoatrR*. The *AoatrR* gene was replaced with a *ptrA* marker in the deletion strain. *Sph*I restriction sites are indicated. The fragment used as a probe is indicated by the black bar. (B) Southern blot analysis to confirm deletion of the *AoatrR* gene. The expected sizes of the bands detected by probing were 5.7 kb and 3.4 kb in WT and *ΔAoatrR* strain, respectively. (C) Genomic structures of *AnatrR* loci in *A*. *nidulans* KU70 and *ΔAnatrR*. The *AnatrR* gene was replaced with a *ptrA* marker in the deletion strain. *Sal*I restriction sites are indicated. The fragment used as a probe is indicated by the black bar. (D) Southern blot analysis to confirm deletion of the *AoatrR* gene. The expected sizes of the bands detected by probing were 11.3 kb and 2.8 kb in WT and *ΔAnatrR* strains, respectively.
(PPTX)

S3 Fig Construction of *atrR* gene deletion in *A*. *fumigatus*. (A) Genomic structures of the *atrR* loci in Af293 and *ΔatrR*. The *atrR* gene was replaced with an *hph* marker (1.35kb) in the mutant strain. *Nsp*V restriction sites are indicated by the blue arrowheads. The fragment used as a probe is indicated as a purple line. (B) Southern blot analysis for checking deletion of *atrR* gene. The expected size of the bands detected by probing were 3.9 kb and 3.6 kb in the WT and *ΔatrR* strains, respectively.
(PPTX)

S4 Fig Drug susceptibility test by paper-disc diffusion assay for the azole fungicides. GMM plates containing conidia of each strain were prepared. A paper-disc was placed on center of the plate, and 10 μl of drug solution indicated was dropped on it (bromuconazole: 10 mg/mL; tebuconazole: 10 mg/mL; difenoconazole: 10 mg/mL; propiconazole: 10 mg/mL). The plates were incubated for 48 h before photographed.
(PPTX)

S5 Fig Characterization of 3x HA-tagged allele of *atrR*. (A) Western blot analysis of AtrR containing a 3x HA tag at its C-terminus. Whole cell protein extracts were prepared from wild-type and *atrR*-3x HA-containing cells. Equal amounts of protein were resolved by SDS-PAGE and then stained with Ponceau S (top panel) to confirm equal loading. This membrane was then subjected to western blotting using an anti-HA mouse monoclonal antibody. The ~100 kDa AtrR-3x HA is only detected in extracts from cells containing the epitope-tagged allele. (B) Azole sensitivity test for the strain containing 3x HA-tagged allele of *atrR*. Spores from the indicated strains were placed on either minimal medium or the same medium containing 0.15μg/ml voriconazole. Plates were incubated at 37°C for 72 hours and then photographed. Note that the strain containing the epitope-tagged *atrR* allele grows at least as well as the wild-type cells confirming that this allele retains function.
(PPTX)

S1 Table AtrR-dependent genes. (XLSX)

S2 Table SrbA-dependent genes. (XLSX)

S3 Table List of primers used in this study. (XLSX)
