# Supplementary material for: A Novel Zn2-Cys6 Transcription Factor AtrR Plays a Key Role in an Azole Resistance Mechanism of Aspergillus fumigatus by Co-regulating cyp51A and cdr1B Expressions
Source: PLoS Pathog. 2017 Jan 4;13(1):e1006096. doi: 10.1371/journal.ppat.1006096 (PMC5215518; doi:10.1371/journal.ppat.1006096)
Supplement: S1 Fig — (A) Amino acid sequence alignment for Zn-finger motifs. Amino acid sequences of the candidate A. oryzae proteins (TF1 to TF5) were compared with that of S. cerevisiae Zn-finger type transcription factors, Pdr1 and Pdr3. The sequence numbers of amino acid in each protein were shown in brackets. (B) Protein motifs in the AtrR proteins. The Gal4-like Zn2-Cys6 DNA binding motif (IPR001138) is indicated by the diagonal line-containing box. The fungal specific sequence motif associated with transcription factor (IPR007219) is indicated by a dotted box. The number above the boxes show the amino acid sequence numbers counted from an initial methionine. (PPTX) [file ppat.1006096.s001.pptx]

## Slide 1
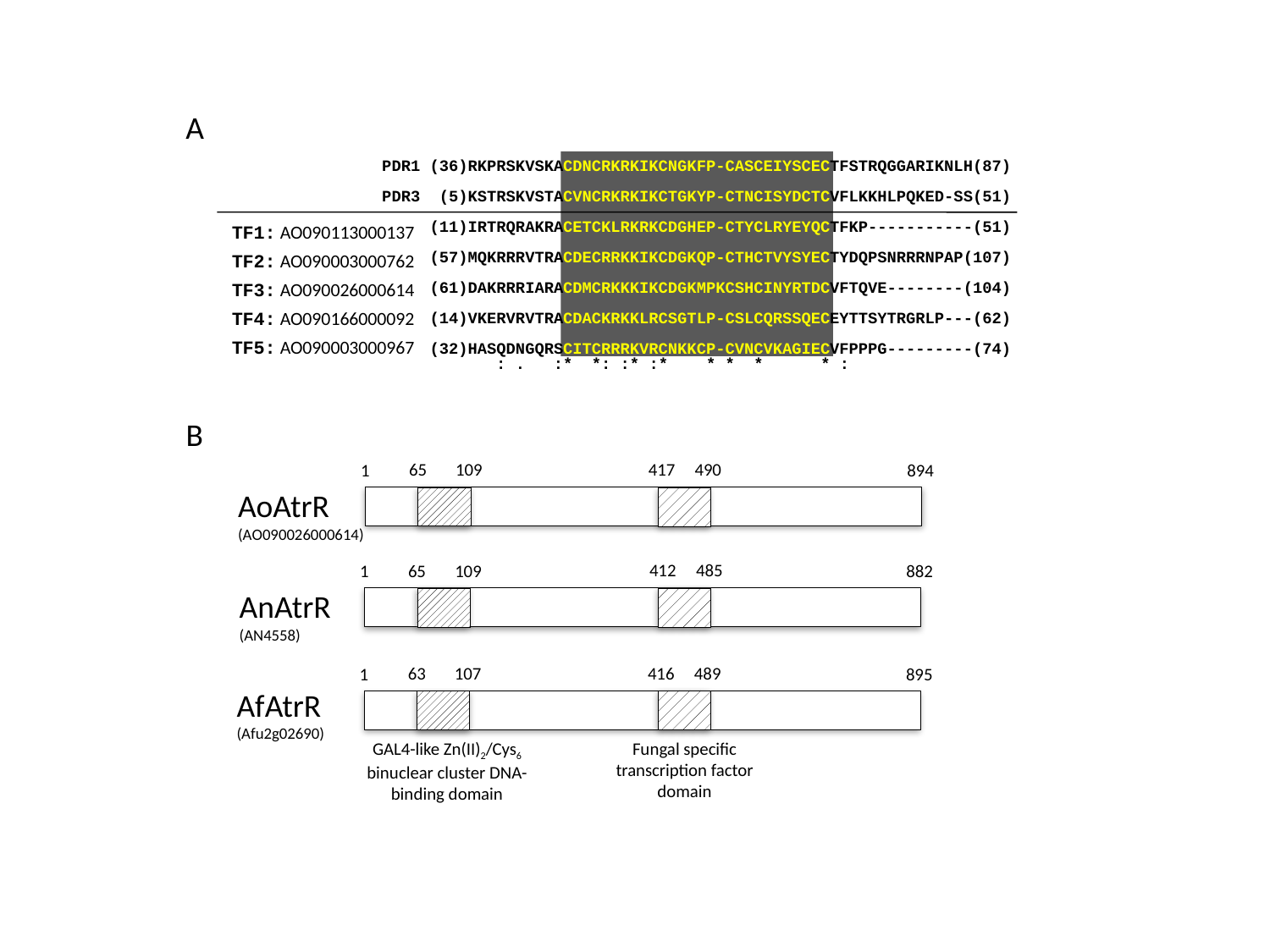

A
PDR1 (36)RKPRSKVSKACDNCRKRKIKCNGKFP-CASCEIYSCECTFSTRQGGARIKNLH(87)
PDR3 (5)KSTRSKVSTACVNCRKRKIKCTGKYP-CTNCISYDCTCVFLKKHLPQKED-SS(51)
TF01 (11)IRTRQRAKRACETCKLRKRKCDGHEP-CTYCLRYEYQCTFKP-----------(51)
TF02 (57)MQKRRRVTRACDECRRKKIKCDGKQP-CTHCTVYSYECTYDQPSNRRRNPAP(107)
TF03 (61)DAKRRRIARACDMCRKKKIKCDGKMPKCSHCINYRTDCVFTQVE--------(104)
TF04 (14)VKERVRVTRACDACKRKKLRCSGTLP-CSLCQRSSQECEYTTSYTRGRLP---(62)
TF05 (32)HASQDNGQRSCITCRRRKVRCNKKCP-CVNCVKAGIECVFPPPG---------(74)
 : . :* *: :* :* * * * * :
TF1: AO090113000137
TF2: AO090003000762
TF3: AO090026000614
TF4: AO090166000092
TF5: AO090003000967
B
417
490
65
109
1
894
AoAtrR
(AO090026000614)
412
485
65
109
1
882
AnAtrR
(AN4558)
63
107
416
489
1
895
AfAtrR
(Afu2g02690)
GAL4-like Zn(II)2/Cys6 binuclear cluster DNA-binding domain
Fungal specific transcription factor domain
