# Supplementary material for: A Novel Zn2-Cys6 Transcription Factor AtrR Plays a Key Role in an Azole Resistance Mechanism of Aspergillus fumigatus by Co-regulating cyp51A and cdr1B Expressions
Source: PLoS Pathog. 2017 Jan 4;13(1):e1006096. doi: 10.1371/journal.ppat.1006096 (PMC5215518; doi:10.1371/journal.ppat.1006096)
Supplement: S2 Fig — (A) Genomic structures of the AoatrR loci in A. oryzae LigD and ΔAoatrR. The AoatrR gene was replaced with a ptrA marker in the deletion strain. SphI restriction sites are indicated. The fragment used as a probe is indicated by the black bar. (B) Southern blot analysis to confirm deletion of the AoatrR gene. The expected sizes of the bands detected by probing were 5.7 kb and 3.4 kb in WT and ΔAoatrR strain, respectively. (C) Genomic structures of AnatrR loci in A. nidulans KU70 and ΔAnatrR. The AnatrR gene was replaced with a ptrA marker in the deletion strain. SalI restriction sites are indicated. The fragment used as a probe is indicated by the black bar. (D) Southern blot analysis to confirm deletion of the AoatrR gene. The expected sizes of the bands detected by probing were 11.3 kb and 2.8 kb in WT and ΔAnatrR strains, respectively. (PPTX) [file ppat.1006096.s002.pptx]

## Slide 1
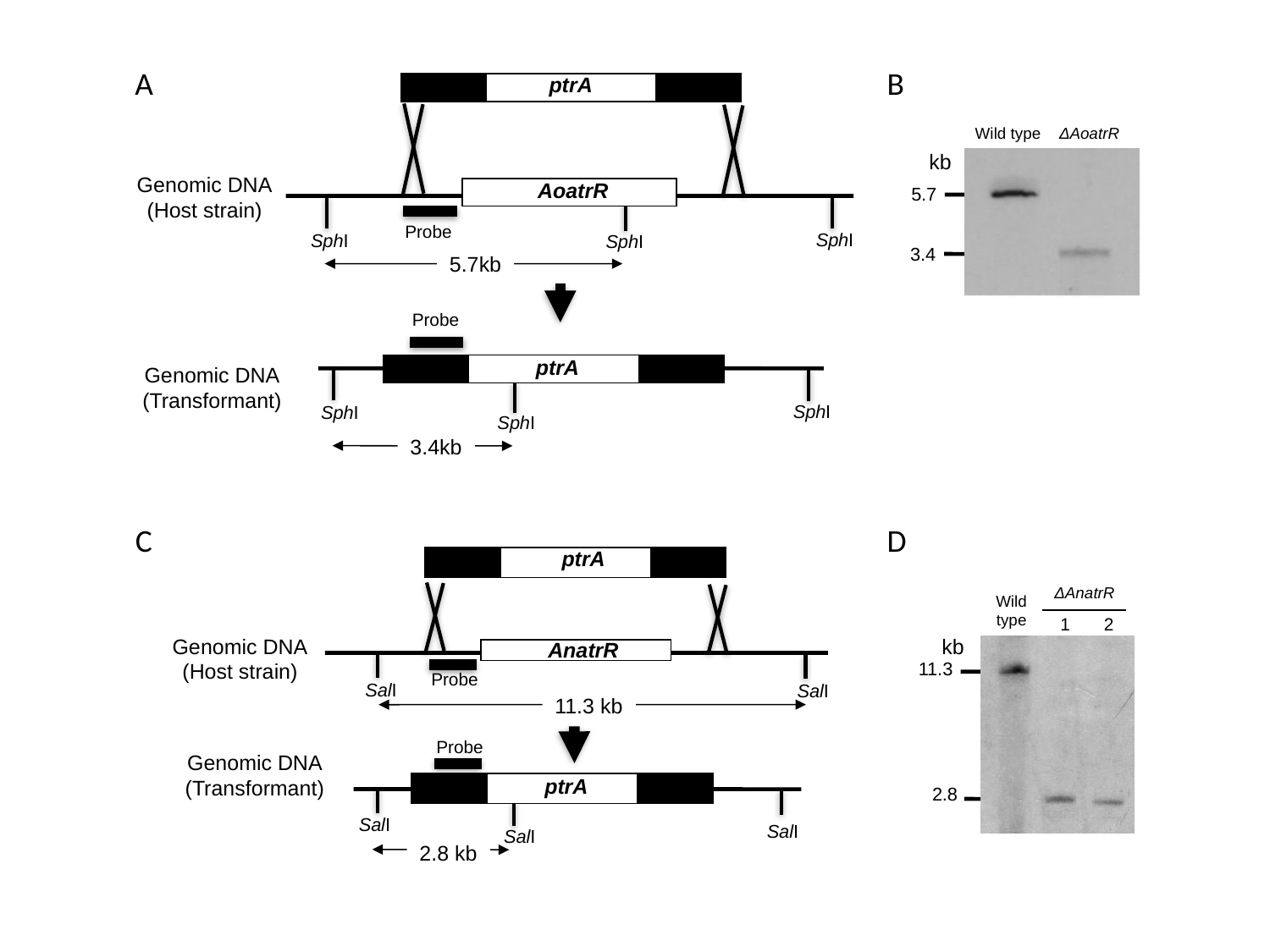

A
ptrA
Genomic DNA
(Host strain)
AoatrR
Probe
SphI
SphI
SphI
5.7kb
Probe
ptrA
Genomic DNA
(Transformant)
SphI
SphI
SphI
3.4kb
B
ΔAoatrR
Wild type
kb
5.7
3.4
C
ptrA
Genomic DNA
(Host strain)
AnatrR
Probe
SalI
SalI
11.3 kb
Probe
Genomic DNA
(Transformant)
ptrA
SalI
SalI
SalI
2.8 kb
D
ΔAnatrR
Wild
type
1 2
kb
11.3
2.8
