# Supplementary figures and images for: A Novel Zn2-Cys6 Transcription Factor AtrR Plays a Key Role in an Azole Resistance Mechanism of Aspergillus fumigatus by Co-regulating cyp51A and cdr1B Expressions
Source: PLoS Pathog. 2017 Jan 4;13(1):e1006096. doi: 10.1371/journal.ppat.1006096 (PMC5215518; doi:10.1371/journal.ppat.1006096)

## Slide 1
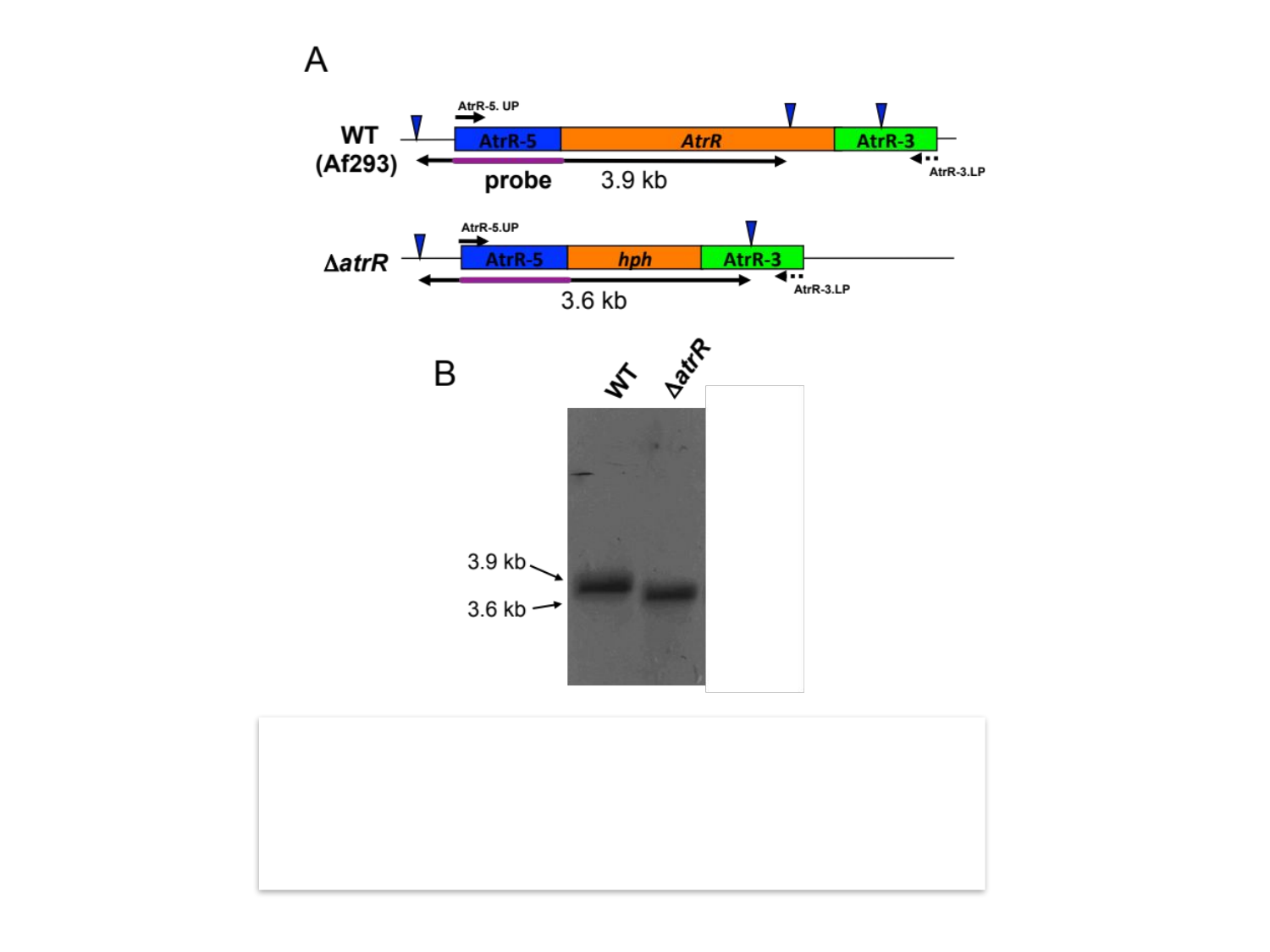

Supplement: S3 Fig — (A) Genomic structures of the atrR loci in Af293 and ΔatrR. The atrR gene was replaced with an hph marker (1.35kb) in the mutant strain. NspV restriction sites are indicated by the blue arrowheads. The fragment used as a probe is indicated as a purple line. (B) Southern blot analysis for checking deletion of atrR gene. The expected size of the bands detected by probing were 3.9 kb and 3.6 kb in the WT and ΔatrR strains, respectively. (PPTX) [file ppat.1006096.s003.pptx]

## Slide 1
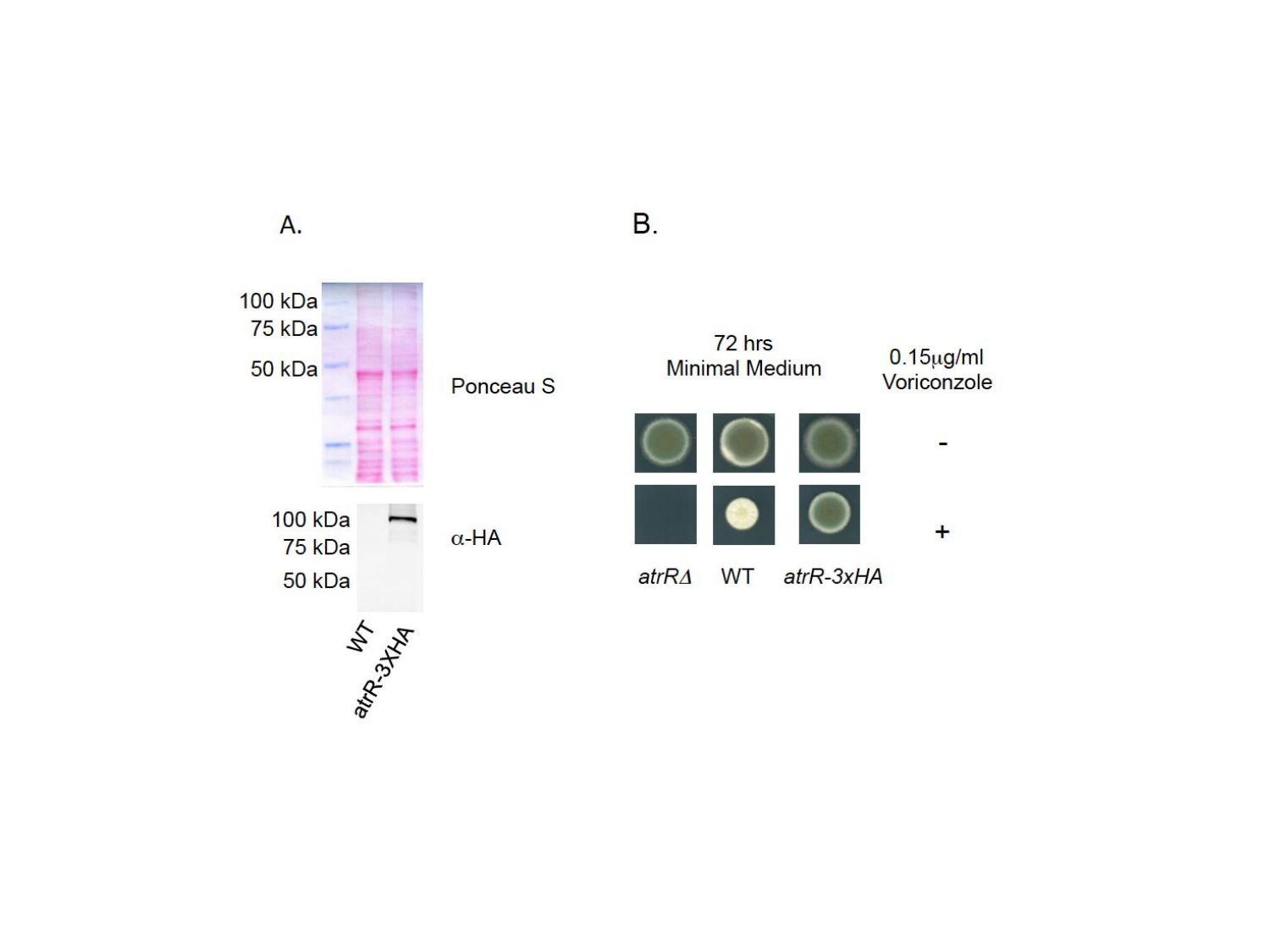

Supplement: S5 Fig — (A) Western blot analysis of AtrR containing a 3x HA tag at its C-terminus. Whole cell protein extracts were prepared from wild-type and atrR-3x HA-containing cells. Equal amounts of protein were resolved by SDS-PAGE and then stained with Ponceau S (top panel) to confirm equal loading. This membrane was then subjected to western blotting using an anti-HA mouse monoclonal antibody. The ~100 kDa AtrR-3x HA is only detected in extracts from cells containing the epitope-tagged allele. (B) Azole sensitivity test for the strain containing 3x HA-tagged allele of atrR. Spores from the indicated strains were placed on either minimal medium or the same medium containing 0.15μg/ml voriconazole. Plates were incubated at 37°C for 72 hours and then photographed. Note that the strain containing the epitope-tagged atrR allele grows at least as well as the wild-type cells confirming that this allele retains function. (PPTX) [file ppat.1006096.s005.pptx]
