# Supplementary material for: A Novel Zn2-Cys6 Transcription Factor AtrR Plays a Key Role in an Azole Resistance Mechanism of Aspergillus fumigatus by Co-regulating cyp51A and cdr1B Expressions
Source: PLoS Pathog. 2017 Jan 4;13(1):e1006096. doi: 10.1371/journal.ppat.1006096 (PMC5215518; doi:10.1371/journal.ppat.1006096)
Supplement: S4 Fig — GMM plates containing conidia of each strain were prepared. A paper-disc was placed on center of the plate, and 10 μl of drug solution indicated was dropped on it (bromuconazole: 10 mg/mL; tebuconazole: 10 mg/mL; difenoconazole: 10 mg/mL; propiconazole: 10 mg/mL). The plates were incubated for 48 h before photographed. (PPTX) [file ppat.1006096.s004.pptx]

## Slide 1
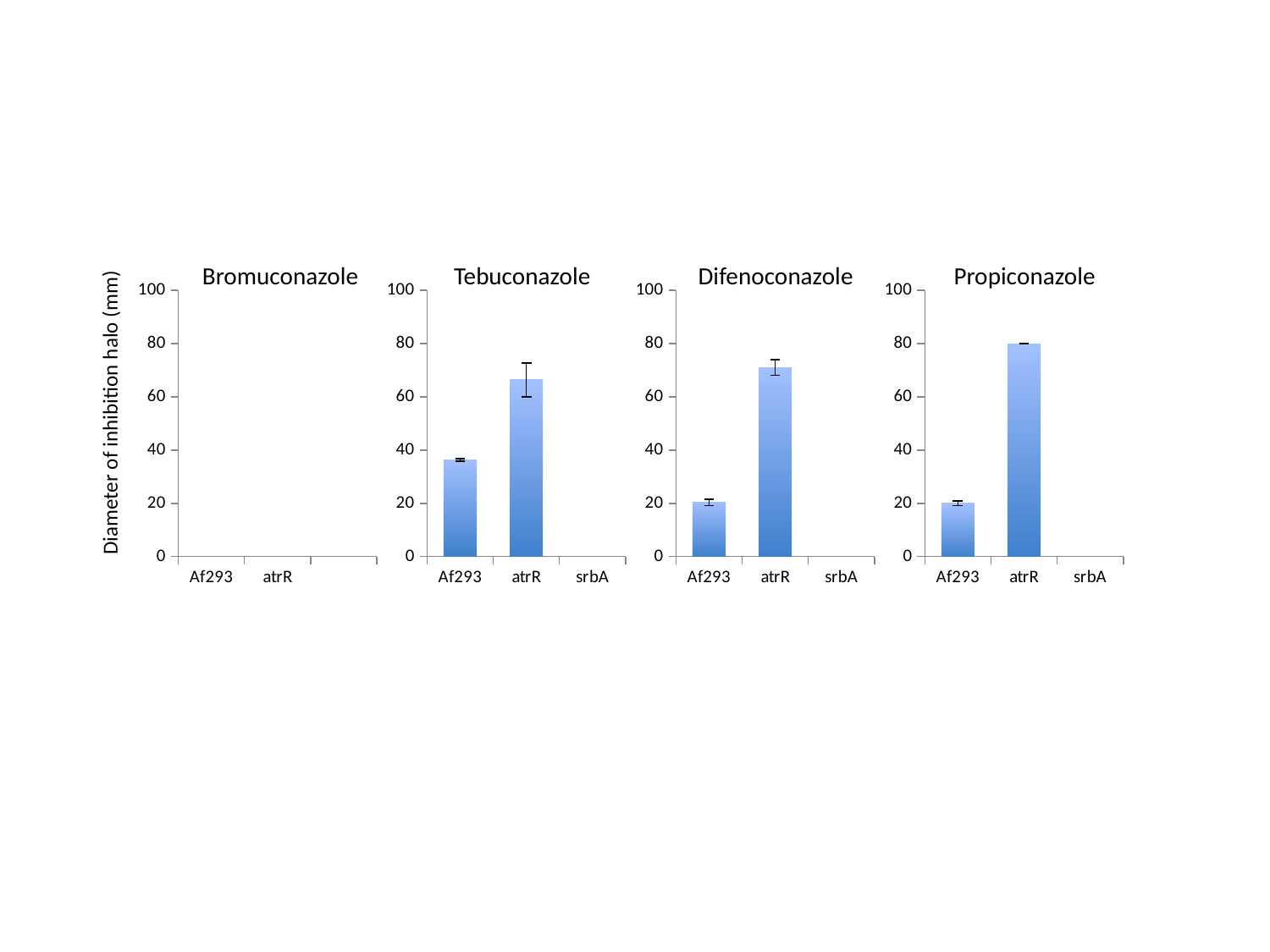

Bromuconazole
Tebuconazole
Difenoconazole
Propiconazole
### Chart
| Category | |
|---|---|
| Af293 | 22.3333333333333 |
| atrR | 80.0 |
[unsupported chart]
[unsupported chart]
[unsupported chart]
Diameter of inhibition halo (mm)
